# Transforming mentorship in STEM by training scientists to be better leaders

**DOI:** 10.1002/ece3.4527

**Published:** 2018-10-02

**Authors:** Amanda K. Hund, Amber C. Churchill, Akasha M. Faist, Caroline A. Havrilla, Sierra M. Love Stowell, Helen F. McCreery, Julienne Ng, Cheryl A. Pinzone, Elizabeth S. C. Scordato

**Affiliations:** ^1^ Department of Ecology and Evolutionary Biology University of Colorado Boulder Colorado; ^2^ Hawkesbury Institute for the Environment Western Sydney University Richmond New South Wales Australia; ^3^ Department of Animal and Range Sciences New Mexico State University Las Cruces New Mexico; ^4^ Department of Veterinary Sciences University of Wyoming Laramie Wyoming; ^5^ Department of Integrative Biology Michigan State University East Lansing Michigan; ^6^ Department of Biological Sciences California State Polytechnic University Pomona California

**Keywords:** leadership, mentoring, professional development, scientific practices, STEM

## Abstract

Effective mentoring is a key component of academic and career success that contributes to overall measures of productivity. Mentoring relationships also play an important role in mental health and in recruiting and retaining students from groups underrepresented in STEM fields. Despite these clear and measurable benefits, faculty generally do not receive mentorship training, and feedback mechanisms and assessment to improve mentoring in academia are limited. Ineffective mentoring can negatively impact students, faculty, departments, and institutions via decreased productivity, increased stress, and the loss of valuable research products and talented personnel. Thus, there are clear incentives to invest in and implement formal training to improve mentorship in STEM fields. Here, we outline the unique challenges of mentoring in academia and present results from a survey of STEM scientists that support both the need and desire for more formal mentorship training. Using survey results and the primary literature, we identify common behaviors of effective mentors and outline a set of mentorship best practices. We argue that these best practices, as well as the key qualities of flexibility, communication, and trust, are skills that can be taught to prospective and current faculty. We present a model and resources for mentorship training based on our research, which we successfully implemented at the University of Colorado, Boulder, with graduate students and postdocs. We conclude that such training is an important and cost‐effective step toward improving mentorship in STEM fields.

## 1 INTRODUCTION: MENTORING IN THE ACADEMIC SYSTEM

Effective mentoring at the graduate and postdoctoral stages in STEM fields (science, technology, engineering, and math) has long been identified as an essential catalyst for performance, success, and career advancement (Eby, Allen, Evans, Ng, & DuBois, 2008; Long & McGinnis, 1985; Lyons, Scroggins, & Rule, 1990; Mullen, Fish, & Hutinger, 2010; Paglis, Green, & Bauer, 2006; Tenenbaum, Crosby, & Gliner, 2001). Indeed, one of the most important factors contributing to how graduate students assess the quality of their educational experience is their relationship with their primary advisor (Austin, 2002; Lovitts, 2001; Lyons et al., 1990; Woolston, 2017). The level and efficacy of support provided by the advisor are also associated with objective measures of student productivity, including number of publications, conference presentations, and interest in research (Cronan‐Hillix, Gensheimer, Cronan‐Hillix, & Davidson, 1986; Lunsford, 2012; Nettles & Millett, 2006; Paglis et al., 2006). Furthermore, effective mentoring can reduce stress, anxiety, and depression (Levecque, Anseel, De Beuckelaer, Van der Heyden, & Gisle, 2017; Panger & Janell, 2014; Peluso, Carleton, & Asmundson, 2011), and positive mentoring relationships have been shown to increase the success and retention of students from underrepresented groups (Brown II, Davis, & McClendon, 2010; Evans & Cokley, 2008; Ortiz‐Walters & Gilson, 2005; Redmond, 2002; Thomas, Willis, & Davis, 2007; Tsui, 2007). By contrast, ineffective mentorship can lead to increased stress and attrition in students as well as decreased productivity and efficiency. This loss of research products and personnel can have cascading negative effects on faculty, departments, and institutions (Burk & Eby, 2010; Eby, Butts, Durley, & Ragins, 2010; Gail Lunsford, 2014; Herzig, 2004; Scandura, 1998).

Despite empirically supported benefits associated with effective mentoring for both the mentee and mentor, academic faculty are generally not given any formal training in mentoring best practices and often do not receive much feedback or supervision from senior colleagues or administrators to help them succeed in leading their laboratory groups. The benefits of effective mentorship offer a clear incentive to invest in formal training to teach best practices and improve mentorship skills and accountability at the graduate, postdoctoral, and faculty levels in STEM fields (Eby et al., 2008; Lunsford, 2012). Although defining an “effective” mentor is challenging due to the individual‐specific nature of the mentoring relationship, mentorship is nonetheless a skill that can be learned and improved with evidence‐based approaches and formal training (Johnson, 2015; Schneider, 2008; Thompson, 2010). In this paper, we outline the unique challenges of effective mentorship in the academic system and present results from a survey of STEM scientists that support both the need and the desire for formal mentorship training among academics. We use these survey results as well as the primary literature to identify characteristics of effective academic mentors. We then provide a model and resources for mentorship training, which we developed and implemented at the University of Colorado, Boulder (CU Boulder). We argue that behaviors and strategies to improve flexibility, communication, and trust can be taught to prospective and current faculty, and emphasize that these trainings can improve the mentorship relationship from the perspective of both mentors and mentees. Finally, we make suggestions for how to improve mentoring at the laboratory group, departmental, and university levels, and discuss the important role of mentoring in improving equity and inclusion in STEM fields.

## 2 MENTORSHIP CHALLENGES IN ACADEMIA

Several features of the academic system create unique mentoring challenges. At research universities, faculty members are often hired and promoted largely based on their successes in research, publications, and funding. However, much of their time as faculty is devoted to teaching, mentoring, and managing a laboratory group—tasks for which they have not necessarily been prepared and for which they may not possess natural talents. Furthermore, the traditional power structures of academic systems make accountability for mentorship efficacy difficult (Huisman & Currie, 2004; Meyer, 2012), and ineffective mentoring behaviors are often met with few consequences, at least over the short term (Higher Education Network 2015). It is difficult for faculty to improve as mentors when they receive little or no feedback. However, students and postdoctoral researchers (postdocs), whose future employment prospects rely on recommendation letters and networking opportunities provided by their mentors, may be reluctant to offer honest or critical feedback on mentorship to their advisors (Burk & Eby, 2010).

A further challenge of academic mentorship is the lack of formal training. In the current system, new faculty are expected to learn how to mentor as they go, often modeling their mentoring style based on experiences with their own mentors (Amundsena & McAlpine, 2009; Lunsford, Baker, Griffin, & Johnson, 2013). This ad‐hoc approach, though sometimes successful, can often result in ineffective mentoring relationships that may derail students’ careers (Eby et al., 2010; Gail Lunsford, 2014; Scandura, 1998). Ineffective mentoring relationships can also hurt early‐career faculty by reducing laboratory productivity before tenure, wasting time and resources, and increasing stress.

Despite these challenges, we believe that mentorship in academia can and should be improved. Instead of using personal anecdotes based on small sample sizes, faculty could learn that there are many approaches to mentoring that have been empirically demonstrated to be successful. This process could, in many ways, parallel the current revolution in STEM teaching at the undergraduate level. The academic community is changing how courses are taught by embracing the use of empirical research to identify best practices, such as student‐centered and active‐learning approaches (Freeman et al., 2014; Love Stowell et al., 2015; Woodin, Carter, & Fletcher, 2010). Teacher training workshops and pedagogy seminars train scientists to implement new teaching methods in their classrooms. Such strategies can apply to mentorship as well, and would be beneficial at several career stages. From graduate students to senior faculty, effective mentorship requires skills that can be learned and improved. We argue that training is particularly beneficial at the graduate and postdoctoral stages to prepare future faculty members for laboratory management and leadership. Furthermore, development of communication and management skills would benefit students pursuing non‐academic careers, thus helping to fill a persistent training gap for PhD students and postdocs who leave academia (Campbell, Fuller, & Patrick, 2005; Cyranoski, Gilbert, Ledford, Nayar, & Yahia, 2011; Fuhrmann, Halme, O'Sullivan, & Lindstaedt, 2011; McGeary, 1995; Woolston, 2017).

## 3 SCIENTISTS WANT MORE MENTORSHIP TRAINING

To explore how scientists view mentoring, assay the availability of mentorship training in academia, and assess interest in more formal mentorship training, we conducted a survey of scientists across STEM fields. We developed an online anonymous survey consisting of eight basic demographic questions and 28 short answer and multiple‐choice questions related to mentoring (see Supporting Information Appendix S5; University of Colorado Institutional Review Board Protocol #16‐0721, Exempt Category 2). We disseminated the survey through listservs such as ECOLOG and evoldir, social media platforms such as Facebook, and targeted emails to STEM departments at major United States institutions. The survey was available from November 23, 2016, through April 14, 2017.

We received 509 responses which we then filtered for completion (>53% completed, all demographic and multiple‐choice questions answered) and geography. All of the incomplete responses were cases where people began filling out demographic questions, which included a “choose not to respond” option, and then stopped taking the survey. We chose to retain only those respondents employed or enrolled in the United States, as this was the largest sample size and American graduate training methods differ from other models (Kehm, 2006). With these requirements, we had 235 responses that we used for analysis. Just over half of the respondents were female (55%). Most were white (82%) and working or training in a biology‐ or environmental science‐related field (63%). Most were trainees: 41% were current graduate (master's or doctorate) students, 18% were postdocs, and an additional 18% were current faculty. Complete survey results are reported in the Supporting Information Appendix S6.

The survey responses reveal several interesting themes. First, academics recognize the negative impact of breakdowns in the mentoring relationship: 70% of self‐described mentees reported that a breakdown had affected their mental health, and 70% said it affected their research productivity. Over half, 58%, of self‐described mentors also said a breakdown had affected their research productivity. Breakdowns in mentoring relationships are also not rare events, with 39% of all respondents experiencing poor mentoring “frequently.” However, 70% of self‐described mentors that took our survey felt that they “rarely” mentor poorly. Thus, among our survey respondents, nearly everyone appeared to recognize the consequences of ineffective mentorship, but mentors perhaps did not recognize their own role in a mentoring breakdown. Although this survey is not exhaustive, this discrepancy may reflect a general lack of communication between mentors and mentees about the state of the mentoring relationship.

Second, many academics do not receive formal mentorship training: 69% of respondents received no formal mentorship training, even among those that spent at least 1 hr/week on mentoring‐related activities. Of those that did receive training, 74% reported receiving only “a little” training. In short responses, that small amount of training consisted mainly of short workshops, seminars, or online trainings. Personal experience as a mentee (54%) and “learning by doing” (57%), rather than formal training, were also chosen as “extremely important” in development of a mentoring style. Furthermore, even though mentorship was selected as the most important factor in graduate school retention and completion rates (68.9% of respondents chose mentoring as “extremely important”), mentoring graduate students was not selected as an important factor influencing faculty hiring and tenure decisions (18% marked mentoring as “extremely important,” compared to 65% who marked “extremely important” for research productivity).

**Figure 1:**
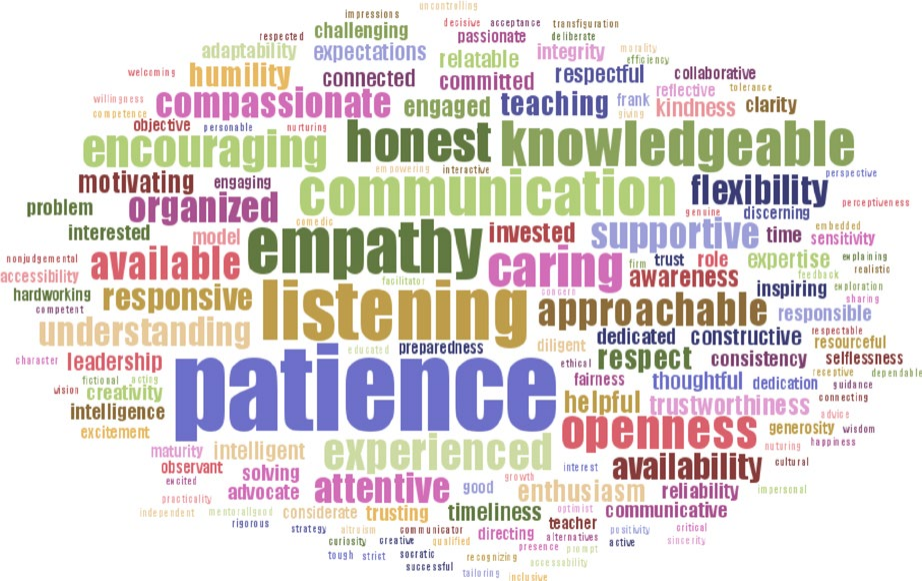
Word cloud showing the results of word‐frequency analysis based on short‐answers to the survey question, “What are three words that describe a good mentor?” The characteristics of good mentors defined by survey respondents, including patience, empathy, and communication, align well with published empirical and popular literature on the characteristics and behaviors of effective mentors and managers

Finally, based on short responses, many respondents think mentors need more training and expressed the desire for more training themselves. A word‐frequency analysis of important characteristics of mentors and mentees revealed broad agreement among respondents: Patience, honesty, listening, and communication were considered top qualities for both mentors and mentees (Figure 1). Training in communication best practices (74%) and conflict resolution (57%) were most frequently selected as activities or resources that would be useful in mentorship training. One respondent summarizes our argument: “Grad students need training in mentoring so that if we become professors [or] bosses we don't suck at it.”

## 4 DEFINING EFFECTIVE ACADEMIC MENTORS

Our survey data clearly indicate that while scientists think mentorship is important and want more formalized mentorship training, there is not currently a broadly implemented framework for mentorship training in STEM fields. The first step in implementing such training is to define effective mentorship. Our survey indicates that people use words like “patience,” “honesty,” “communication,” “empathy,” and “listening” as characteristics of good mentors. Here, we take these impressions further by examining the primary literature to develop a definition of effective mentorship appropriate to the academic setting.

Defining effective mentorship is challenging in part because of the many different roles that the primary mentor (typically the “academic advisor”) must assume for undergraduates, graduate students, and postdocs (Jackson et al., 2003; Jacobi, 1991; Mertz, 2004). The tasks of the academic advisor fall into several categories. First, they provide direction, set standards for work in their field, and help mentees develop and troubleshoot their research projects (Vilkinas, 2008). Second, the advisor is the principal gateway for mentees into their departmental and discipline communities, and therefore needs to advocate for mentees and help them establish connections with other colleagues (Bair, Grant Haworth, & Sandfort, 2004; Barnes & Austin, 2009; Krauss & Yasukawa, 2013; Lovitts, 2001; Lyons et al., 1990). Third, the advisor must identify when students are struggling and help them find solutions (Barnes, Williams, & Stassen, 2012; O'Meara & Braskamp, 2005). Finally, advisors must manage and mediate conflicts between members of their laboratory groups and between themselves and mentees. The best advisors strive to model excellent scholarship and provide sponsorship, collaboration, and encouragement to build student skills and confidence (Barnes, Williams, & Archer, 2010; Lunsford et al., 2013; O'Meara, Knudsen, & Jones, 2013).

Despite these varied mentorship roles, effective mentors exhibit several common behaviors in their interactions with mentees. We place these universal behaviors of effective mentors into three categories: flexibility, communication, and trust. Key behaviors of effective mentors cited by survey respondents fit well within this framework. For example, “patience,” “honesty,” “accessibility,” “respect,” and “listening” were listed as important behaviors of effective mentors (Figure 1). “Listening,” “accessibility,” and “respect” are key components of good communication; “patience” is a component of flexibility; and “honesty” and “respect” are certainly critical to building trust. These categorizations are also consistent with the “popular” literature on management and leadership used in the private sector. For example, popular books on leadership and teamwork emphasize open, honest communication and trust as critical to effective teams and managers (Lencioni, 2002; Patterson, 2012; Stone, Patton, & Heen, 2000). These same themes are also reflected in the available academic literature on effective mentoring and laboratory leadership (Barker, 2010; Guberman, Saks, Shapiro, & Torchia, 2006). Although some people are, for example, naturally better communicators than others, all of these skills build on one another and can be learned and improved. We believe that good mentors are defined by their actions and interactions rather than their innate personalities.

### 4.1 Flexibility

An effective mentor must be able to adapt mentoring strategies to the needs of different students and to a single student over time (Judith & Garrett, 1991; Rose, 2005). Across their careers, mentors will work with students who represent a range of personality types, working styles, backgrounds, preferences, and experiences (Barnes et al., 2010; Rose, 2003). Individual students also change as they progress through their careers, and the relationship with their mentor needs to evolve as well. A diversity of viewpoints, backgrounds and experiences improves creativity and productivity within groups (Hong & Page, 2004; McLeod & Lobel, 1992; Paulus & Brown, 2007), but it also presents a challenge for the person responsible for mentoring a wide range of students. Developing flexibility in mentorship requires mentors to communicate with students and pay attention their individual needs and progress mentors should also have a variety of mentoring strategies and tools at their disposal that enable them to adjust their approach to different students (O'Meara et al., 2013; Opengart & Bierema, 2015). This is particularly important for mentoring and supporting students from groups typically underrepresented in STEM fields (Box 2). Formal training can expand this toolkit (see Supporting Information for resources) and can improve the communication necessary for mentors and mentees to set clear and mutually agreed upon expectations and goals for the mentoring relationship (see next section).

### 4.2 Communication

Taking the time to structure and practice honest, positive, and open communication is key for establishing clear expectations, mutual respect, and shared values among mentors and mentees (Barnes & Austin, 2009; Lovitts, 2004; Nettles & Millett, 2006). Clear and frequent communication with a mentor has been shown to improve student confidence and motivation during graduate school (Eller, Lev, & Feurer, 2014; Nakamura, Shernoff, Hooker, & Csikszentmihalyi, 2009). When mentors are accessible and mentees feel comfortable communicating openly, conflicts can be resolved more quickly, mentors can more effectively help students through difficult periods, and student progress is more efficient. When mentoring relationships fail, poor communication practices are typically a root cause (Barker, 2006; Eby et al., 2010; Herzig, 2004; Scandura, 1998). Routine communication should therefore be established early in mentoring relationships to build rapport, resolve misunderstandings, and make difficult conversations easier in the future. This is particularly important for students who may not be comfortable initiating communication with mentors on their own (see Box 2). Communication skills can be taught and improved, and there are many available resources and training programs which can easily be implemented in academia (e.g., Nakamura et al., 2009; Pfund, Maidl Pribbenow, Branchaw, Miller Lauffer, & Handelsman, 2006; Stone et al., 2000, see Supporting Information for resources). Communication requires effort and investment from both parties, and mentors trained in communication best practices can subsequently help mentees improve their communication skills.

### 4.3 Trust

Trust in mentoring relationships is a crucial factor in student success and retention (Eller et al., 2014; Handelsman, Pribbenow, & Lauffer, 2005; O'Meara et al., 2013). Trust takes time and patience to develop, but is ultimately essential to the establishment of a collaborative, productive, and mutually beneficial relationship between mentor and student. Mentors can build trust with their mentees by being honest and transparent when communicating, working to reduce fear and intimidation when interacting with students, always guaranteeing and maintaining confidentiality, admitting and apologizing for mistakes, and being reliable and consistent in demands, expectations, and promises (Handelsman et al., 2005; Nakamura et al., 2009). Additionally, studies show that trust can be improved when mentors encourage students to take the lead in conversations about goal‐setting, and when mentors always maintain ethical and professional behavior when interacting with mentees (Beres & Dixon, 2016; Fleig‐Palmer & Schoorman, 2011; O'Meara et al., 2013; Waldeck, Orrego, Plax, & Kearney, 1997). Trust is hard to earn and easy to lose, particularly in relationships with a power imbalance. Mentors should be vigilant about avoiding behaviors that may lose the trust of their mentees. Mentees also have an important responsibility in building trust, which can be met by being reliable, honest, and open with their mentors. When trust is established between mentor and mentee, it enables the communication necessary for students to provide honest feedback to their mentors without fear of repercussions (Bozeman & Feeney, 2008; Fleig‐Palmer & Schoorman, 2011). Listening and responding positively to feedback is one of the best mechanisms by which mentors can both improve and maintain trust with their mentees.

## 5 THE WAY FORWARD: A MODEL FOR IMPROVING MENTORSHIP IN STEM

We implement our definition of effective academic mentoring—characterized by the development of flexibility, communication, and trust between mentor and mentee—within a formalized training framework. We propose a model where mentorship training occurs at the graduate and postdoctoral level. This is an ideal career stage to receive mentorship training for several reasons: (a) Many graduate students and postdocs already mentor undergraduates and other less experienced researchers; (b) this training will prepare graduate students and postdocs who intend to become faculty before, rather than after, they take on critical mentoring responsibilities; (c) graduate students and postdocs may have more time to devote to mentorship training than new professors, who are faced with a barrage of new tasks; and (d) mentorship training will benefit graduate students and postdocs who do not intend to become academic faculty, as nearly any career requires skills in leadership, communication, and conflict management (Campbell et al., 2005; Cyranoski et al., 2011; Fuhrmann et al., 2011). However, we note that all faculty could benefit from this training model.

Managing and mentoring students and postdocs in the laboratory parallels management in other fields, particularly the technological sector (e.g., “knowledge workers”). In contrast to academia, companies in the private sector have long recognized that good management practices increase employee productivity and retention and have consequently made management training a priority (Bozionelos et al., 2016; Kantor, 2015; Kram & Hall, 1989; Pololi et al., 2016; Rampton, 2015; Weese, Jakubik, Eliades, & Huth, 2015). Although rewards in academia differ from industry, the reward structures are similar: Rewards in academia are publications, grants, and accolades, as opposed to bonuses, raises, and profit margins. Models and techniques developed for management and leadership in the private sector can therefore be adapted to the academic context. These resources offer tools for individual, team‐based, and peer mentoring approaches that can be applied to scientists (e.g., Ammeter & Dukerich, 2002; Duhigg, 2016; Jakubik, Eliades, & Weese, 2011; Masalimova, Schepkina, Leifa, Shaidullina, & Anatolyevna, 2014; Moon, 2014; Underhill, 2006). Although mentorship in academia is often individual‐based, building trust and communication among groups of collaborators and laboratory members is crucial for scientific progress. With all of these resources available, academics do not need to develop their own training programs de novo. We therefore looked to both the academic literature and the corporate world to develop a training model for academic mentorship.

We implemented this training model at CU Boulder as a semester‐long seminar attended by both graduate students and postdocs (for course syllabus, see Supporting Information Appendix S3). This seminar has been offered twice: once in spring 2016, with nine participants, and again in spring 2017, with 10 participants. The seminar was offered for graduate credit and met for 1.5 hr each week for 14 weeks. It has been highly successful: Participants report feeling substantially more prepared and comfortable in mentoring roles, and the training has qualitatively improved existing mentoring relationships between participants and their mentees, as well as between participants and their own advisors. We also condensed parts of this seminar into shorter formats: (a) a 2‐hr presentation given to first‐year graduate students, with the goal of providing them with resources for developing a positive relationship with their advisors based on effective communication and (b) a 1‐hr presentation given in a faculty, graduate student, and postdoc seminar on diversity in STEM, which covered mentoring strategies for recruiting and supporting students from underrepresented groups. Additionally, we distilled the main points from the training seminar into a departmental mentoring “best practices” document (available in the Supporting Information Appendix S1), which has been adopted by the Department of Ecology and Evolutionary Biology at CU Boulder.

In the training seminar, we emphasized developing skills and techniques for building trust, communication, and flexibility in mentor–mentee relationships, as well as a clear understanding of mentoring best practices and ethics. To help participants develop these skills, we used case studies, role‐playing, written exercises, discussions, and readings (available in the Supporting Information Appendices S3 and S4). Learning goals and activities for the course fall into the following categories: (a) defining effective mentorship and identifying mentoring best practices, (b) exploring mentoring personality and preferences, (c) practicing effective mentorship in conflict situations, (d) understanding professional behavior and ethics in the context of mentoring relationships, and (f) synthesizing participants’ mentoring philosophy into a mentoring statement. We briefly discuss each of these categories and provide example seminar content, below. Additional information, including a course syllabus, readings, activities, and other resources, is included in the Supporting Information.

### 5.1 Identifying best practices and defining effective mentorship

We began the seminar by having participants list their “gut instincts” of qualities that make a good mentor, as well as list what they perceive to be mentorship challenges based on their own experiences. We used these lists as a starting point for developing a more formal mentorship framework. Participants read and discussed existing STEM literature on mentoring and laboratory management (Barker, 2010; Guberman et al., 2006; Handelsman et al., 2005). This literature suggested several concrete best practices for academic mentoring, including having regular meetings between mentors and mentees, setting goals, and avoiding inappropriate behavior. However, it largely lacked a discussion of how to develop the underlying fundamentals of effective mentoring relationships (e.g., mentoring strategies for different students, and how to build flexibility, communication, and trust).

To fill this gap, we drew from management resources developed in the private sector and consulted with experts from academic and non‐academic fields (see Supporting Information Appendix S4). This broader literature offered recommendations on how to develop an effective mentoring relationship. It also provided a better framework for managing conflicts and mentoring a diversity of people. We adapted these solutions for STEM‐specific mentoring relationships. For example, we used feedback from the CU Boulder Faculty Affairs Office to develop a guide for writing professional emails, which are the source of many professional conflicts and miscommunications. We also outlined how to run more effective and productive meetings by setting agendas and cutting time‐wasting activities, following guidelines developed by companies such as Google and Apple. We identified strategies used to hire personnel and build effective teams in the tech sector and applied them to recruiting graduate students and postdocs and managing laboratory groups, collaborations, and field crews. We discussed how to establish clear expectations and motivate mentees to do their best work, while still building trust and support. Details of all of these topics are in Supporting Information Appendix S3. Many of these techniques were drawn from four widely recommended resources (Lencioni, 2002; Patterson, 2012; Regier, 2017; Stone et al., 2000). We began and ended each of the seminar meetings by modeling tools developed in an executive management context to build trust, improve communication, and provide honest feedback among seminar participants (see “check‐ins” and “plus delta” in the Supporting Information Appendix S3).

### 5.2 Explore mentoring personality and preferences

An important first step to becoming an effective mentor is to clarify one's own personality and preferences as they relate to information processing, communication, and working style, and to understand how differences in these preferences among mentees may lead to mentoring challenges. To explore personality types and preferences, each participant took the Myers–Briggs Type Indicator (MBTI) Step II personality test (Furnham, 2017) and the StrengthsFinder 2.0 evaluation (Rath, 2007 ). Experts at the CU Boulder Career Services center administered these evaluations and facilitated discussion of the results. Participants spent several sessions exploring results of the evaluations. This included small group work to examine communication, responses to stress, and conflict management in the context of personality differences. We broadly discussed how these personality differences influence mentorship in the context of one‐on‐one interactions and teamwork. These evaluations provided participants with a shared vocabulary for discussing differences in working styles, communication preferences, and responses to stress. We acknowledge that such personality tests have limitations and that retesting does not always yield consistent results (Michael, 2003; Pittenger, 2005). However, we found that the exercise of identifying personality and preference differences, and exploring how people with similar or different personality types interacted, was very useful in the context of mentoring (Rowe, 2017). The MBTI is not the only metric that could be used to accomplish these goals (see the Big‐Five factor inventory: Goldberg, 1990; or Hexaco personality inventory: Lee & Ashton, 2004). This component was widely reported to be the most illuminating aspect of the seminar with respect to improving mentoring relationships.

### 5.3 Practicing effective mentorship in difficult situations

Many of the activities in the mentoring seminar focused on building practical mentoring skills. Participants worked on applying methods from outside STEM to academic mentoring scenarios. Often, this took the form of case studies: Participants took turns playing the role of a mentor in pairs or small groups and responded to hypothetical, challenging mentoring scenarios. For example:

Your graduate student wants to delay their candidacy exam because they don't feel ready, but you want them to have it this semester and you think that they will do fine. What do you say?

You set a deadline to review a paper that your graduate student is writing. You did not hear anything from your student for a week prior, and now the deadline has passed. What do you do?

You feel like something is wrong in the personal life of your grad student (they have seemed tired and stressed lately and slow on email). What do you do?

Additional examples are included in the Supporting Information Appendix S3. The person role‐playing the mentor would respond either through verbal communication or email, and explain their approach. Their partner(s) would then explain how they would react as the “mentee.” The group would then discuss a variety of possible solutions to the mentorship scenario. For these case studies, we paired participants with different personality types and preferences and frequently switched partners, highlighting the fact that an approach that works for one individual may be ineffective for another. These case studies allowed participants to see how their specific choices as a mentor may impact mentees, while also providing examples of different strategies to solve common problems and increase flexibility.

### 5.4 Professional behavior and ethics

Throughout the seminar, we discussed professional behavior in the context of mentoring relationships, as well as ethical issues that may arise in academic mentoring. These issues are very important in academia, where mentoring relationships are typically long term and interactions often take place outside the traditional workplace. The close nature of these relationships can often blur professional and personal boundaries (Beres & Dixon, 2016), and mentors may therefore forget that there is a power hierarchy inherent to these relationships that leaves mentees vulnerable (Burk & Eby, 2010; Eby et al., 2010). We discussed how to maintain professional boundaries in mentoring relationships, while still building trust and support. We also discussed basic guidelines for professional behavior when interacting with mentees (e.g., sending appropriate emails, providing objective feedback, not losing your temper, respecting your mentee's privacy). Additionally, we discussed ethical concerns related to intellectual property, authorship, letters of recommendation, bias in selecting and providing opportunities to students, and confidentiality. Professional behavior and ethics are of particular importance when conflict arises (Lynch, 2017), so we invited a professional executive coach to discuss conflict resolution in the seminar.

### 5.5 Express a mentoring philosophy

The final course assignment was to write a mentoring philosophy. With this assignment, trainees were asked to synthesize what they learned in the seminar into a document that would be useful in the future. In preparation for writing mentoring statements, we convened multiple faculty panels, in which participants had the chance to ask current faculty members about their mentoring style, experiences, and challenges. Some participants then wrote mentoring statements to be included with a teaching statement in future faculty job applications, some wrote laboratory mentoring guides that would be shared with their future mentees as they advanced to faculty positions, and others wrote mentoring philosophies with specific best practices that could be used in existing mentoring relationships. During the last week of the seminar, participants conducted peer reviews of each other's statements, while reflecting on the skills gained in the course.

Participant feedback indicated that our training model at CU Boulder has been successful at providing practical knowledge and mentoring skills within a formal framework supported by empirical data. Supporting such mentorship training is a low‐cost way to invest in the future of academic STEM researchers. In addition to providing mentoring training, academic departments and institutions can promote effective mentoring in other ways. Effective and inclusive mentoring benefits not only the people being mentored, but, through increased retention and productivity, also benefits departments and institutions at large. Solutions at the institutional level are discussed further in Box 1, and the specific impacts of positive mentorship on recruitment and retention of students from underrepresented groups are discussed in Box 2.

BOX 1 **DEPARTMENTAL AND INSTITUTIONAL SUPPORT FOR MENTORING**

1 In the main text, we focus on the steps both mentors and mentees can take to build a healthy and productive one‐on‐one mentorship relationship. However, mentoring does not take place in a vacuum, and top‐down approaches that target training, social climate, and culture at the departmental and institutional levels will also play a key role in improving mentorship (Keyser et al., 2008; Petridis, 2015). As we highlighted with the survey data, if mentoring is not valued at higher administrative levels, mentors may have little incentive to improve and mentees may not feel that their voices are heard. Given the discipline‐specific nature of graduate training (Hirt & Muffo, 1998), we advocate for the development of department‐ specific mentoring resources and best practices guidelines. The following are suggestions for administrators seeking to improve mentoring in their department and institution.

**Department level**

- Collect anonymous data on the social climate and current mentoring practices within the department
- Develop mentoring guidelines that lay out clear expectations for the graduate mentor–mentee relationship and describe evidence‐based best practices. Allow students as well as faculty to have a voice in the development of these guidelines
- Provide informal mediation for mentors and mentees seeking to improve communication
- Facilitate mentoring workshops for faculty and students
- Disseminate departmental and campus resources for conflict resolution
- Discuss diversity issues such as implicit biases before student admission and hiring decisions

**Institution**

- Collect anonymous data on the campuswide social climate and mentoring practices across departments
- Require training in mentorship and diversity sensitivity for all faculty
- Recognize and reward effective mentorship in retention, tenure, and promotion decisions
- Facilitate collaboration between faculty across departments for improving mentoring
- Provide counseling and conflict–resolution services for faculty and students
- Improve career counseling tailored specifically to graduate students
- Recognize and reward outstanding mentors

BOX 2 **UNDERREPRESENTATION IN STEM AND THE ROLE OF EFFECTIVE MENTORING**

1 The underrepresentation of numerous minorities in STEM fields is an ongoing problem that has received much recent attention (Gibbs, 2014; Briggs, 2016; Jones, 2016; Malcom & Malcom‐Piqueux, 2013; Morris & Washington, 2017). The positive effects of equitable representation of minorities on scientific research go beyond inclusivity: extensive evidence shows that diverse groups are more creative and better at solving problems (Hong & Page, 2004; McLeod & Lobel, 1992; Paulus & Brown, 2007). People with a diversity of perspectives, experiences, and approaches are critical for science to keep pace with the many emerging problems in our changing world.

Mentoring has a crucial role to play in supporting and retaining members of underrepresented groups in STEM. We emphasize that the mentorship approach and best practices that we have laid out in this paper benefit all students and faculty, regardless of background. However, for students who are members of underrepresented groups, poor mentoring has been shown to be disproportionately detrimental and contributes to attrition rates (Estepp, Velasco, Culbertson, & Conner, 2017; Kendricks, Nedunuri, & Arment, 2013; Ortiz‐Walters & Gilson, 2005; Park‐Saltzman, Wada, & Mogami, 2012; Thomas et al., 2007), whereas good mentoring can be disproportionately beneficial to recruitment, support, and retention (Brown II et al., 2010; Cox, Yang, & Dicke‐Bohmann, 2014; Evans & Cokley, 2008; Kendricks et al., 2013). We argue that improving mentorship is an important step in addressing issues of underrepresentation in STEM.

In developing a positive and productive mentoring strategy to enhance inclusion and equity in STEM and recruit and retain a diverse workforce, the universal rules apply: flexibility, communication, and trust. Flexibility is particularly important as students from backgrounds underrepresented in STEM may have different expectations for their mentors (Blake‐Beard, 2009; Brown II et al., 2010; Chan, 2008; Dedrick & Watson, 2002). Specifically, power distance, or the extent to which individuals are comfortable with disparities in the distribution of power, varies significantly between cultures and countries, as well as across individuals (quantified as the power distance index (Davidson, 2001; Cox et al., 2014)). Students who perceive a large power distance are often less comfortable initiating communication with their mentor and may prefer formal rather than informal communication (Richardson & Smith, 2007). Mentors may need to make a special effort to establish transparent and honest communication, build trust, and adapt their mentoring style to the needs and expectations of students who come from a background that is different from their own (Chan, 2008; Girves, Zepeda, & Gwathmey, 2005; Wilson, Andrews, & Leners, 2006). If the mentor makes a genuine effort to understand the motivations of their mentee, cultural differences can be a resource, not a gap (Blake‐Beard, 2009; Park‐Saltzman et al., 2012).

### 5.6 Extensions: Assessment of mentoring

While we were unable to include a formal assessment beyond general reflection and discussion with the participants in our seminar, we believe that evaluative assessment will be an important tool for making real improvements to mentorship in STEM fields. Effective mentoring is valued outside of academia because there are clear and measurable benefits associated with good leadership and tangible rewards for those leaders. To make changes to academic mentorship culture and improve accountability, an objective assessment program that rewards effective mentors, provides honest and constructive feedback, and requires improvement is essential. Rewards that require extra work (e.g., nominating someone or applying for a mentorship award) may be attractive to only a subset of people. Instead, mentorship performance and student success should be a critical component of hiring, tenure review and promotion decisions and should inform decisions about which faculty have the opportunity to take on new students.

There are important challenges to consider when evaluating mentorship. Effective mentoring can be difficult to measure as it involves both metrics of graduate student success (e.g., publishing papers, progress toward graduation), and less tangible metrics such as student mental health, support, and satisfaction. Additionally, evidence from research on teaching evaluations suggests that students can be biased when evaluating certain groups, such as women, and thus evaluations based on student opinion alone must be used with caution (Boring, 2017; Mengel, Zölitz, & Mengel, 2017; Miles & House, 2015; Stark & Freishtat, 2014; Storage, Horne, Cimpian, & Leslie, 2016). Finally, finding mechanisms that allow for honest feedback in academia is challenging, as graduate students and postdocs must be protected from retribution in cases of a negative review.

Despite these challenges, we feel that evaluating mentorship and providing guidance for improvement, as well as a structure for accountability, are important steps forward in changing the culture of how mentorship is valued in STEM fields. Evaluations will need to be carefully designed and executed to address potential biases. Similar concerns surrounding teaching evaluations have led to the development of new metrics and evaluation techniques that better measure student success and are less prone to bias (Golding & Adam, 2016; Gormally, Evans, & Brickman, 2014; Miller, 2015; Wieman, 2015; Winchester & Winchester, 2014). Similar tools could be adapted for mentorship evaluations. Assessment methods will need to take a holistic view of student–faculty relationships. For tenure or promotion decisions, emphasis should be placed on evaluating patterns of successful or ineffective mentoring across several students instead of focusing on isolated incidents, although isolated incidents should be appropriately dealt with and used as learning experiences. We suggest that annual reviews, where both mentors and mentees report anonymously to an impartial third party about how their relationships are functioning, may be a step in the right direction. In this case, feedback can be given as a summary to each person with clear information about where they are doing well and areas that need improvement, paired with opportunities for training. Routine assessment and constructive feedback would increase transparency and help detect and resolve problems early, which would benefit both students and faculty. Furthermore, funding agencies that require mentoring statements for postdocs and graduate students as part of grant proposals (e.g., the National Science Foundation) could solicit feedback from mentors and mentees to evaluate the efficacy of the mentoring relationship at the conclusion of a project. Accumulated negative feedback could impact future funding decisions. This is similar to the adoption of Broader Impact standards.

## 6 CONCLUSION

Effective mentoring has demonstrable impacts on productivity, creativity, inclusion, equity, and mental health, and thus, effective mentoring should be a central goal of STEM faculty members, departments, and institutions. There is no doubt that most scientists want to be effective mentors, yet they receive little to no training and often lack essential skills for accomplishing this goal. Despite these challenges, we believe that as a community we should make mentorship a priority, and, in doing so, make substantive improvements by providing scientists with the skills they need to be effective mentors. Our proposed training model is an effective and affordable step in the right direction. It seeks to improve mentorship by providing formal training to graduate students and postdocs, who are the future leaders and mentors of STEM fields. This training draws on strategies and best practices from the corporate world and from empirical academic research. We have successfully implemented this model at CU Boulder, where it is now expanding to other STEM departments. We encourage other institutions to establish and support similar courses.

## AUTHOR CONTRIBUTIONS

All authors participated in the first iteration of the mentoring seminar and contributed to writing and editing the formal document and supplement. AKH and ESCS developed and led the original mentoring seminar and completed a majority of the writing. AKH reviewed a majority of the academic mentoring literature and compiled the majority of the Supporting Information. ESCS and HFM led the second mentoring seminar. HFM compiled the description of the mentorship model for this manuscript. SMLS created the survey and analyzed the results. ACC and CAH conducted literature reviews of mentoring practices outside of academia. JN, CAP, and AMF contributed to compiling and organizing the Supporting Information. Authors other than the first and last are listed alphabetically.

## DATA ACCESSIBILITY

All questions, results, and raw data from the mentoring survey are presented in the Supporting Information for this manuscript, Appendices S5 and S6.
