# Supplementary material for: Transforming mentorship in STEM by training scientists to be better leaders
Source: Ecol Evol. 2018 Oct 2;8(20):9962–74. doi: 10.1002/ece3.4527 (PMC6206201; doi:10.1002/ece3.4527)
Supplement: Supplementary file 1 [file ECE3-8-9962-s001.docx]

***Appendix S1***

**Example Departmental Best Practices in Graduate Mentoring Document**

***Department and University Name***

The role of the advisor is diverse and may include several areas of guidance, advising, support, and supervision. The advisor is a role model for students, helping them learn norms and ethics in academic behavior and performance at the levels of the department, university, and the broader field of study. The best mentors strive to model how to engage in excellent scholarship, provide sponsorship, collaboration, practical supervision, and encouragement to build student skills, and confidence as they progress through graduate school, and beyond.

Each advisor develops their own mentoring style based on personality, working style, and experience. While mentoring styles may vary, we encourage faculty to recognize that there are well defined, and empirically supported, tools, strategies, and approaches that work. These best practices can be learned, practiced, and adapted to improve the mentoring experience and outcomes for both the student, and the mentor. In addition, it is important to recognize each student is unique and thus the style of mentoring that best suits a student's needs will vary. Students will also change over time, and adjustments in advising will be necessary as a student’s progress leads to increasing responsibilities and independence. Students should recognize that their capacity and responsibilities will increase as they advance in graduate school and that an important role of their advisor is to push them and challenge them to help them build their abilities in order to meet their goals. Advisors should recognize that graduate students are doing and learning many things for the first time, they will make mistakes, and will learn to be more efficient and effective with their work through time.

Start early in the mentoring process and establish the norms for communication, expectations, and requirements. This can be an important part of interviewing new graduate students, benefiting both faculty and student. When students are deciding to join a lab after acceptance, they should have a clear idea of the type of mentoring and support they will receive. We encourage faculty to develop their own written mentoring statement or contract that they discuss with prospective students and new graduate students. This conversation should include 1) determining the best ways for the advisor and student to communicate with each other, and how frequently they will meet. 2) Discussing what the expectations of the advisor for the student are in terms of performance (e.g. chapter/ manuscript preparation), and what the student expects from the advisor in terms of assistance and advice (e.g. help with thesis topic, reading the literature, comments on drafts) in making progress toward their degree. It should also touch on 3) working styles and preferences of both the mentor and student, and what type of mentoring or feedback works best for the particular student, and 4) the student’s short and long-term goals and the role of the advisor in helping them to reach those goals. It is important to regularly revisit this conversation and expectations with each student as needs will change over time. Faculty and students should recognize that openly discussing potential areas of conflict (e.g. authorship, financial support, differences in working styles, research expectations and timelines), early and often, before problems arise, is the best way to avoid conflict and maintain a good and productive mentoring relationship.

We encourage advisors to facilitate these discussions with each of their students every semester, or at least once a year. Advisors and students should review together the requirements for obtaining the degree, including coursework, exams, teaching, and documents (thesis, dissertation) (seeDepartmental or University regulations for reference). Establish a strategy for setting goals, evaluating progress, and identifying challenges on a regular basis (e.g., weekly or monthly). Both advisors and students should understand their rights and responsibilities, as outlined in the Professional Rights and Duties of Faculty Members (*link to your department document*) and the Graduate Student Bill of Rights and Responsibilities (*link to your departmental document*).

**Best Practices: How to enhance mentoring and the graduate student experience:**

|  | **Graduate students are encouraged to:** | **Faculty mentors are encouraged to:** |
| --- | --- | --- |
| **Establishing mentoring**  **relationship norms** | Meet with faculty advisors during their first semester to:   - Discuss how often and in what manner they will communicate to share progress and concerns; regular meetings with your faculty advisor are strongly encouraged - Identify effective strategies for regular communication so that you can receive feedback and direction as needed. - Discuss any expectations for presence on campus while working (e.g. expected to work in the lab during certain hours or days)   Meeting your mentoring needs:   - Your advisor may not be able to provide you everything you are looking for in a mentor. This is okay, and expected. - Work to establish a mentoring network to get the help and support you need. Form relationships with other faculty, postdocs, and graduate students. | When working with a new student:   - Facilitate discussions about mentoring norms, communication, expectations, and goals. - Consider how differences in personality and preferences will affect your working relationship with this student - Explain the support network available to the student and their role in the student’s education (e.g., labmates, postdocs, committee members, EBIO staff, cohort).   Foster a healthy research community by:   - Providing graduate students with ways to directly and honestly communicate their concerns and needs for assistance. - Providing encouragement and constructive feedback on student progress |
| **Working Logistics** | Determine the expectations of their advisor and committee for:   - The formulation of a research project, - Execution of the research - Final presentation of the advisory committee (exams, defense) - Expectations for publication - Notice needed for feedback on drafts, or letters of recommendation - Expectations for regular meetings with committee members | Share with students:   - Your expectations of student progress and timelines - Your expectations for their research topics, if any - The steps and time management needed to successfully execute a thesis or dissertations, teach effectively and participate in service and outreach - Details of the support you can provide for research and funding. - Clear expectations for what is expected from students for receipt of that support |
| **Career Advancement** | Discuss with their advisors and committees   - Career goals, and the best ways to achieve and be prepared for those career goals. - Areas of concern, or particular challenges - Areas where they would like to grow, topics or skills they would like to develop or learn. - Opportunities they should pursue to meet their goals (grants to apply to, courses to take, collaborations, conferences) and how to prioritize these opportunities with other responsibilities and requirements - Seek advice on applying to and securing desired future positions to meet career goals | - Discuss and understand the student’s long-term goals and incorporate appropriate training opportunities into the student’s education plan (e.g., teaching, research, outreach). - Encourage students to read the scientific literature thoroughly and frequently - Help students to enhance their writing and speaking skills - Encourage and support students to attend meetings and seminars. - Facilitate networking and professional development opportunities - Discuss possible career tracks, and suggest appropriate courses and opportunities   **Be aware that student goals should supersede faculty goals for the student.** |
| **Dissertation progress** | - Early in a student’s graduate program, they should review the requirements of the degree and determine a timeline for meeting those requirements - Discuss progress towards degree requirements with their advisor on a regular basis, at least once a semester. - Emphasize possible bottlenecks, or trouble spots, where help may be most needed. | - Work with students to establish reasonable goals for the completion of milestones that need to be achieved in order to complete their degree. - Help student to overcome possible barriers to meeting these goals and to grow and learn from these experiences - Be clear about your expectations for lead time to receive drafts for comments, and for letters of recommendation. |
| **Conflict Resolution** | - Bring up areas of potential conflict and stress before they become a problem - Open communication can prevent conflicts, so keep your advisor in the loop - Prevent misunderstandings by asking questions and clarifying expectations if things are unclear - Send professional emails - Maintain professional and respectful behavior at all times - Use available resources and bring in help to resolve conflicts if needed. | - Discuss, and provide opportunities for students to bring up, areas of potential conflict and stress before issues arise - Make sure expectations and timelines are clear - Maintain open communication - Discuss small problems before they build to larger problems - Send professional emails - Maintain professional and respectful behavior at all times - Use available resources and bring in help to resolve conflicts if needed. |

**Worst Practices: things to avoid doing:**

- Remaining silent when inappropriate behavior is observed (may be governed by the University’s policy on discrimination and harassment)
- Losing your temper, sharing confidential information, and other unprofessional behavior
- Holding grudges, or not allowing for growth, as students make mistakes and learn during graduate school
- Neglecting or ignoring your student for extended periods of time
- Using unreasonable threats in order to get students to complete assignments or goals.
- Placing a student in the middle of a dispute, including disputes between faculty members or between other members of the lab group.
- Pressuring students to do things they are uncomfortable doing that are not required as part of their education. These could include personal favors or work unrelated to university-related teaching or research.
- Encouraging unhealthy behavior (e.g. excessive drinking, lack of sleep, work-life imbalance).
- Taking student ideas, data, or research (intellectual property), without appropriate credit, permission, and collaboration.
- Putting your goals for the student above the goals of the student themselves.
- Having inconsistent or unclear demands and expectations of students.
- Discouraging students from asking questions, clarification of expectation, or seeking help.
- Discriminatory behavior, or treating students differently because of gender, race, status, etc., or fostering an environment that is not inclusive to a diversity of students.

**University Resources Available for Assistance**

***(Insert relevant information for your department)***

Professional Rights and Duties of Faculty Members:

Graduate Student Bill of Rights and Responsibilities:

University’s policy on discrimination and harassment:
Counseling and Psychiatric Services:

Graduate School Resources:

Ombuds Office:

Graduate Advising:

Grievance Policy:

*This department guide was developed using resources from the University of Minnesota mentoring program and feedback from the University of Colorado Ecology and Evolutionary Biology Department faculty.*

***Appendix S2***

**Example lab-level mentoring agreement**

This is an outline of suggested topics to cover in a lab-level mentoring agreement. As a PI, we would encourage you to think carefully about your answers to each of these topics. You don’t need to write down an answer to every question, but having a clearly articulated mentoring approach and agreement is a useful resource for both current and prospective lab members. We would also encourage PIs to develop a mentoring document with all lab members. In an ideal world, this mentoring agreement would be a “living document” that evolves over time and incorporates input and feedback from all lab members. By having everyone invest in such a document, communication channels can be cleared and conflicts/ miscommunications avoided.

**Statement of lab and mentoring philosophy**:

Outline a mentoring philosophy. What are your goals as a mentor? What kind of lab environment do you strive to create?

*Example:*

In this lab, we value integrity, hard work, communication, creativity, trust, and collaboration; these are the foundations of effective scientific inquiry. We endeavor to create a lab environment where all members are free to share and discuss their ideas and where trust and respect are paramount. Our goal is to catalyze exciting scientific research by providing a constraint-free environment where people with diverse perspectives and a common passion for biology can interact positively and productively. (*adapted slightly from John Orrock’s lab at Wisconsin*)

Within this broader lab environment, my goal as a mentor is to help you (as my mentee) to identify and work towards your goals, whatever they may be. I will do my best to ensure that our relationship is based on mutual trust and respect, open and honest communication, and adaptability. Research is nothing if not the relentless pursuit of better solutions, and I encourage feedback at any time on how to improve our mentoring relationship and the lab in general.

Lab members represent a diversity of races, genders, backgrounds, and sexual orientations. We are united by a shared respect for the scientific process and a shared interest in producing high-quality, exciting research. Anyone that is dedicated to pursuing excellent science and is respectful of other people is welcome here.

**General expectations**

What are your expectations of your lab members?

Are projects generally collaborative, or do different students have their own, independent projects?

How many hours a week do you expect lab members to be in lab?

How will you assess progress?

Will you monitor how your mentees spend their time?

At what rate do you expect progress to occur?

**Funding, authorship, etc.**

You may want to set separate expectations for different groups (i.e. undergraduates, graduate students, postdocs).

Are lab members responsible for obtaining their own funding?

What contributions warrant authorship on papers?

Will you as the PI automatically be the senior author on papers coming out of the lab? How many thesis chapters/papers are generally expected in a particular time frame? Can students take data/projects with them when they leave the lab?

How much time can be spent working on projects from previous positions?

How much outreach/service do you expect lab members to participate in?

**Mentoring approach and communication**

Will there be regular one-on-one meetings? If so, how often?

Do you expect that you and your mentees will set goals together?

How do you expect to communicate with your lab members?

Are you hands-on or hands-off?

How often do you expect updates on projects?

What types of decisions do you expect lab members to make on their own, vs. what decisions should be checked with you first?

What do you expect students to prepare for one-on-one meetings?

How often will you hold one-on-one meetings?

**Email etiquette and expectations**

How quickly do you expect lab members to respond to emails?

Do you expect responses outside of normal working hours?

Do you prefer to discuss issues over email or in person?

Do you expect emails to be formal or informal?

What are your opinions on exclamation points and emojis?

**Lab presence and maintenance**

Do you expect everyone to be in the lab during normal working hours, or are flexible schedules permitted?

How many hours a week do you expect people to be in the lab?

Who is in charge of lab maintenance- cleaning, ordering supplies, etc?

What are policies regarding food, music, and pets?

**Lab meetings format and expectations**

How often are there lab meetings?

Is attendance mandatory?

What is the format of lab meetings?

**Conflict mediation**

How will you deal with conflict if it arises?

What type of feedback do you want from lab members?

What resources are available for conflict mediation?

***Appendix S3***

**Example Mentoring Course Syllabus**

**Mentoring and Leadership in Science (Course Number)**

**University Name, Semester and Year**

**Instructor Names**

**Learning Goals and Objectives:**

Mentoring students and managing personnel are critical skills for scientists. However, most graduate students and postdocs never receive any formal training in management, mentorship, or leadership. This may result in new faculty frequently feeling out of their depth and falling back to an ad-hoc mentoring approach based on their own personal experiences. This course aims to fill this gap in training.

There is a massive amount of empirical literature that shows effective mentoring and management increase student and employee productivity and wellbeing. This has long been known in the private sector, where companies such as Google spend tremendous time and money to identify what management practices will best increase employee productivity, creativity, and efficacy of teamwork. These data show that good management and mentoring are skills that can be learned, practiced, and improved.

In this course, we will draw on tools and techniques developed in the private sector and social science, and apply them to mentoring students and managing personnel in STEM. Even if you don’t plan to remain in academia, good leadership and management skills are broadly applicable, and will be useful as you pursue a career in industry or other fields. The course is structured as a combination of discussion, readings, invited speakers, and various interactive exercises. We will begin the semester with sessions aimed at identifying and developing personal mentorship and leadership styles and philosophies. We will then move on to conflict management, team building, and practical mentoring and management skills such as running efficient meetings and sending good emails. We will address, discuss, and practice such topics as writing and reviewing letters of recommendation, talking through conflict scenarios, avoiding common pitfalls, and adjusting mentorship style for individuals with different backgrounds and personality types.

**Product:**

One product of this seminar will be for each participant to produce a 1-2 page document that outlines their personal mentorship philosophy. The format and content of these statements may vary, but we recommend structuring them in such a way that they can be discussed and easily referenced with mentees.

**Recommended Readings:**

1. *Making the Right Moves: A Practical Guide to Scientific Management for Postdocs and New Faculty*, Burroughs Wellcome Fund and Howard Hughes Medical Institute
2. *The Five Dysfunctions of a Team*, Patrick Lencioni
3. *Difficult Conversations: How to Discuss What Matters Most*, Douglas Stone, Bruce Patton, Sheila Heen.
4. *At the Helm, a Laboratory Navigator,* Kathy Barker
5. *Entering Mentoring: A Seminar to Train a New Generation of Scientists:* Howard Hughes Medical Institute Professors Program, Jo Handelsman, Christine Pfund, Sarah Miller Lauffer, Christine Maidl Pribbenow.

**Class Schedule**

**Class 1: Introduction to the course**

Start with short presentation explaining why mentoring is so important.

1. Introduce “check-ins” and "plus delta”

- A tool for building trust in groups and generating constructive feedback

- - A chance to mimic the techniques you may want to apply to your own research group

1. Introvert-extrovert exercise
   - Where are you on this scale? What does that mean for how you interact with others in a work environment?
2. Discussion questions:
   - What are your biggest questions about leadership and mentorship?
   - What makes a good/bad mentor?
   - What do you hope to get out of this course? How will our time be effective?
3. **Assignment**: Pre-assessment questions to answer
4. What are your biggest strengths and weaknesses as a mentor?
5. What do you want to work on over the semester?
6. Read chapter 5 in the HHMI mentorship manual

**Class 2: Mentoring strengths and weaknesses**

1. Partner up and discuss your strengths and weaknesses from the assignment
2. Discuss as a group to identify common strengths, weakness, and general issues to address during the semester
3. Short presentation of mentoring tips based on HHMI chapter 5
4. **Assignment:** online MBTI test (often provided through University)

**Class 3: Case studies on mentoring scenarios**

1. We have developed 5 different mentoring case studies. These examples focus on setting expectations, fostering independence, and building trust
2. Pair up and discuss how you would handle each scenario
3. After 5 minutes of discussion in pairs, we will discuss our solutions as a group.
4. **Assignment**: finish online MBTI test by next meeting

**Class 4: Myers-Briggs Type Indicator results**

1. Class will be led by an MBTI facilitator from the University
2. **Assignment**: read “MBTI and Conflict” documents

**Class 5: Conflict management in the context of MBTI**

1. This class will be led by an MBTI facilitator

**Class 6: Conflict management- application of new techniques to case studies**

1. Break into groups corresponding to your MBTI type (more similar together)
2. We provide case studies of common sources of stress or conflict in mentor-mentee relationships
3. Discuss how you would respond to each stressful scenario within your subgroup. Make a list of common themes/responses.
4. As a class, share and discuss how people with different MBTI grouped types would respond to each conflict/stress scenario.
5. **Assignment**: read document on good email practices

**Class 7: Best practices for email and meetings**

1. How to run productive and efficient meetings, based on techniques used at Google, Apple, and other tech companies that employ knowledge workers
2. Best email practices presentation
3. Pair up with someone with a different MBTI type. Write an email in response to the conflict scenarios presented last week, and discuss how different types would react to your phrasing
4. **Assignment**: fill out the StrengthsFinder quiz online (through University)

**Class 8: StrengthsFinder facilitation with University Career Services**

1. This class will be led by a University facilitator from Career Services
2. **Assignment:** read *Student performance measures that don’t perform* (Kuo, *Science* 2017); *Recommendation letters reflect gender bias* (Kuo, *Science* 2016).

**Class 9: Hiring and Team Building, Invest in your Values Assessment**

1. Read two example letters of recommendation as a class. Discuss how they would influence hiring decisions.
2. Presentation on interview strategies: what to look for, what questions to ask, how to hire the best people, how to avoid implicit bias
3. Invest in your Values Assessment tool: determine what motivates your team members (working independently, having high income, being part of a team, gaining new knowledge, etc)
4. **Assignment**: send us discussion questions for the faculty panel next week

**Class 10: Discussion with faculty panel-** junior faculty

1. Discussion will focus on how faculty members have developed their mentoring styles, as well as challenges they have faced and suggestions for young researchers

**Class 11: Discussion with faculty panel**- senior faculty

1. Continuation of questions from previous week
2. **Assignment**: finish your personal mentorship statement, read statements from two other course participants

**Class 12: Mentorship statements**

1. Small group discussion and feedback of mentoring statements
2. **Assignment**: read through departmental faculty advising guide

**Class 13: Departmental advising guide**

1. Class discussion to evaluate and improve the departmental advising/mentoring document

**Class 14: Wrap-up / Reflections over the semester**

*We have provided some examples and resources from the course, specifically those referenced in the main manuscript, below. You are welcome to contact us and request any additional resources mentioned in the course description.*

**Liz Scordato:** Elizabeth.Scordato@colorado.edu

**Amanda Hund:** Amanda.Hund@colorado.edu

**Helen McCreery:** Helen.McCreery@colorado.edu

**Example Case Studies and Discussion Questions:**

1. Your graduate student just got rejected for a grant and isn’t sure if they can finish their experiment. You don’t have money to cover any of their project expenses. What conversation do you have?
2. You set a deadline to review a paper that your graduate student is writing. You did not hear anything from your student for a week prior, and now the deadline has passed.
   1. It is their independent project
   2. You are the senior author on the paper and need it published soon

1. You were really stressed last week and sent a terse email to your graduate student about how he/she needs to let you know more than a week in advance if they need a letter of recommendation from you (you were asked 3 days ahead of time). Now you feel bad about the email and that maybe it came off too harsh.
2. The undergraduate students that your grad student supervises are leaving a mess in the lab. What do you do?
3. You feel that something might be wrong in the personal life of your graduate student or postdoc (they have seemed tired and stressed lately and slow on email). What do you do?
4. Your graduate student wants to delay their candidacy exam because they don’t feel ready, but you want them to have it this semester and you think with some preparation, he/she will be fine. What do you say?
5. Your volunteer field assistant has slept in for the 3rd morning in the past two weeks. This is holding up the rest of the team. You can’t fire the person midway through the season, what do you say?
6. You are relying on volunteer field assistants to collect important field data. You need this information to set up your experiment. They are frequently late at sending/ entering the data, which is making it really hard for you to organize your experiment. What do you say?
7. One of your graduate students is less involved in optional lab activities than the others, like getting dinner or a drink after work, because of their parenting activities for their young son. Although their work is good, they aren’t as integrated into the lab as you would like and haven’t developed a rapport with the lab group. How do you help include them in the broader lab culture?
8. Power distance mismatch: You’re a new assistant professor and you’ve been mentoring a graduate student for a few months. They interact with you very informally, joking around, and they frequently challenge you and occasionally miss deadlines. It’s important they feel comfortable and that they can come to you with concerns, but sometimes you think they don’t have enough respect for the fact that you often need to be the one making final decisions. How do you discuss these concerns?
9. You’re mentoring an undergraduate research assistant, who is a first-generation college student and has no previous research experience. You’re frustrated by having to explain things that your other, more experienced students already knew before they started working with you. You want to be supportive and help your student succeed, but you’re very busy and have project deadlines to worry about. How do you deal with the situation?

**For additional case studies see: The Howard Hughes Medical Institute Professors Program: *Entering Mentoring A Seminar to Train a New Generation of Scientists***

<http://www.hhmi.org/sites/default/files/Educational%20Materials/Lab%20Management/entering_mentoring.pdf>

**Group Exercises (plus delta, check-ins)**

As a way to model exercises that could be used with a lab group or larger collaboration we began and ended each of our seminar meetings by modeling tools developed in executive management context that are explicitly designed to build trust, improve communication, and provide honest feedback. These include “check-ins,” in which each trainee mentioned a recent professional success and challenge, and a recent personal success and challenge, and a “plus-delta” exercise, wherein each person states something that they thought went well and something that could be improved about the meeting.

**Summary Email Guide**

**General considerations before sending an email**

- What is your relationship with the person you are emailing?
- What time are you sending this email?
- How will the recipient interpret the timing of the email?
- When do you expect a response?
- How detailed of a response do you expect?
- What tone do you expect- formal? informal?
- What issue are you discussing? Is it straightforward or complex?

**Email etiquette**

1. ***Assess your relationship with the person to whom you are sending a message.*** If you have a strong working and personal relationship with your recipient, he or she will be more likely to understand your words as you intend them. If you don’t, be exceedingly careful in what you write.
2. ***Treat your message as you would a formal memo.*** Draft. Revise. Have someone else read your draft. Don’t send a substantive email at night. Sleep on it.
3. ***Before sending, consider how you’ll feel if your message is forwarded to others,*** including your department colleagues. There is no such thing as a confidential email.
4. ***If your email prompts a negative response, don’t send another email explaining what you meant or justifying yourself.*** Allow yourself only one round of email. Thereafter arrange to meet in person (or over skype if necessary) to clear things up.

**Sending Good Emails**

- Make sure the point of the email is very clear
- Be Concise
- Proofread
- Be as “reader friendly” as possible. Use headings, bullet points, etc. to break up the text.
- Subject line should concisely convey the main point or takeway of your email.

**Before Sending, ask yourself:**

1. Is this message suitable for e-mail, or could I better communicate the information with a phone call or face-to-face meeting?
2. What is my purpose for sending this e-mail? Will the message seem important to the receiver, or will it be seen as an annoyance and a waste of time
3. How many e-mails does the reader usually receive, and what will make him/her read this message (or delete it)?
4. Do the formality and style of my writing fit the expectations of my audience?
5. How will my message look when it reaches the receiver? Is it easy to read? Have I divided my thoughts into discrete paragraphs? Are important items, such as due dates, highlighted in the text?
6. Have I provided enough context to easily understand the message?
7. Did I identify myself and make it easy for the reader to respond?
8. Will the receiver be able to open and read any attachments?

**Be a good email responder**

1. Read your emails
2. If an email requires a detailed response that will take you a few days, let the sender know
3. Respond to all the points that the sender has brought up

**Tone:** To avoid misunderstandings, read your message out loud before hitting send. If it sounds harsh to you, it will sound harsh to the reader. For best results, avoid using unequivocally negative words ("failure," "wrong," or "neglected"), and always say "please" and "thank you.”

**Email Style Discussion Points:**

1. How to greet?
2. How to end?
3. Use of exclamation points
4. Humor or sarcasm
5. Grammar and spelling

For fun, check out: [www.crystalknows.com](http://www.crystalknows.com)

**Summary Meeting Guide**

**Seven Steps to Run a Good Meeting**

1. Make your Objective Clear
2. Consider who is invited, does everyone need to be there?
3. Stick to your schedule
4. Take no hostages
5. Start on time, end on time
6. Ban Technology
7. Follow up

**Advice from the Howard Hughes Medical Institute**

- Start each meeting with agenda / plan
- Send a follow up summary and to-do list after meetings
  - Use minutes to start next meeting
  - Help gauge progress and keep track of tasks and ideas

**Advice from Apple**

- Attendees should walk away with concrete next steps / actions
- Every project or task should have a DRI (directly responsible individual)
- Be prepared to challenge and be challenged
- Answer is always: yes and, and never no, but

**Advice from Google**

- No more than 10 people
  - Invite as few as possible
- Focus: kill ideas and meetings when you can
- Keep it short and productive
- Review homework from last meeting to track progress

***Make every meeting matter - or don’t meet at all!***

**When not to hold a meeting**

- Key person can’t make it
- Agenda or readings have not been distributed enough in advance
- Purpose or goal is unclear
- Work can be done faster in another way
  - email, phone, individual meetings

**Meeting Killers**

- Wasting meeting time
- Wasting people’s time
- Boring meetings that go nowhere
- Meetings for meeting’s sake
- Tangents

**Hold a workshop, not a meeting**

- ⅓ problems
- ⅓ solutions
- ⅓ tasks and priorities


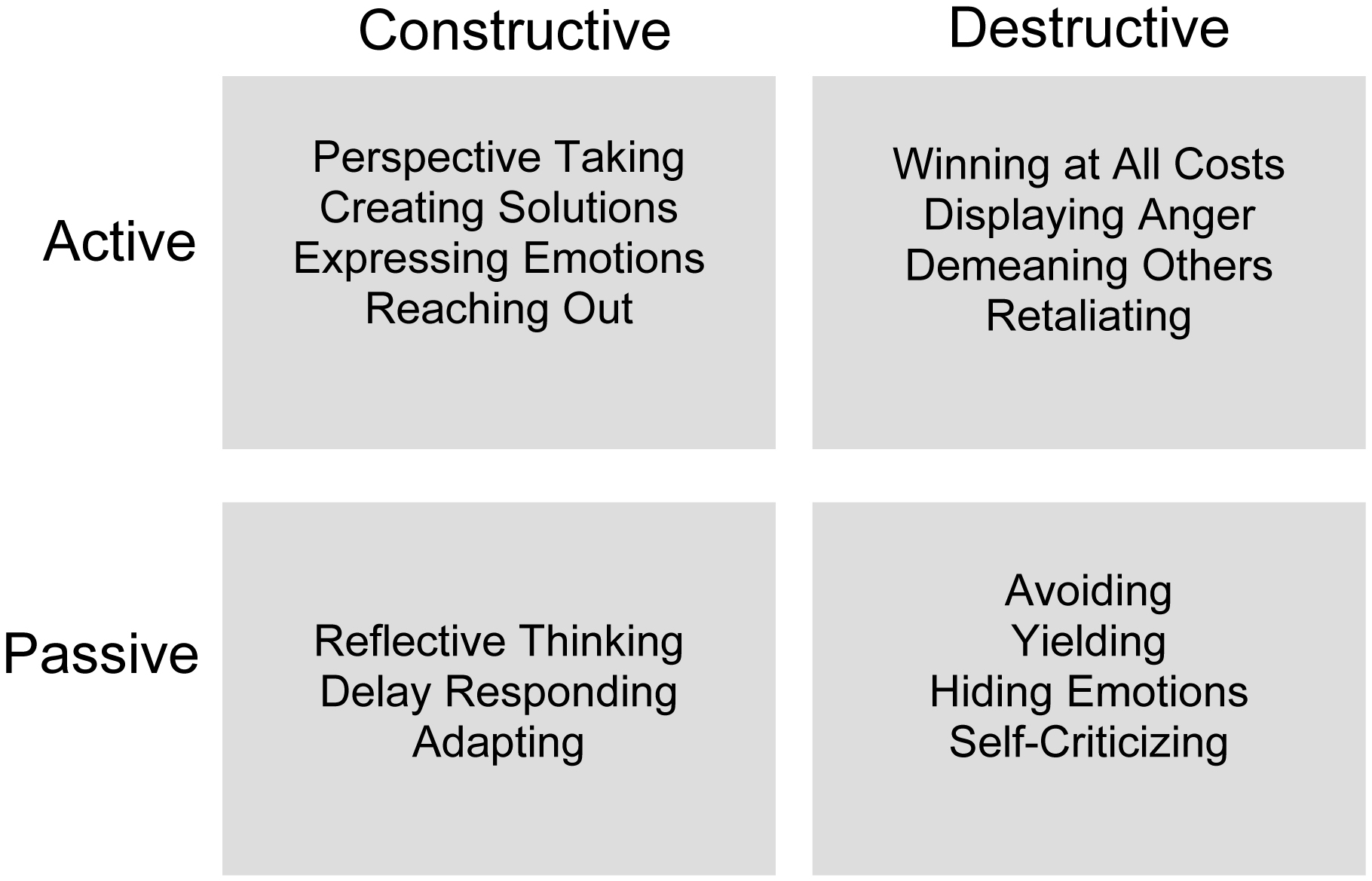


Figure used when discussing good meeting behaviors and how to recognize poor meeting behaviors. *Adapted from Balancedcurve and Xponents.*

***Appendix S4***

**MENTORING RESOURCES**

***Graduate Mentoring Guides***

Following are some examples of mentoring guides that have been implemented at various academic institutions.

- The University of Michigan’s Rackham Graduate School has developed both faculty and student guides on mentoring. This guide, which was first produced in 1999, has been continually revised throughout the years and has been adopted and adapted by a number of universities across the United States.
  - Link: <http://www.rackham.umich.edu/mentoring>
  - Examples of schools that have adapted the Rackham Graduate School mentoring guide:
    1. University of Washington: <http://grad.uw.edu/for-students-and-post-docs/core-programs/mentoring/>
    2. University of Nebraska Lincoln: <http://www.unl.edu/mentoring/>
- The Howard Hughes Medical Institute has written a downloadable book specifically for postdocs and new faculty that includes a chapter on being both a mentor and mentee.
  - <http://www.hhmi.org/developing-scientists/making-right-moves>
- Emory University, Laney Graduate School has developed both a faculty and student guide to building successful mentoring relationships:
  - Faculty Guide: <http://www.gs.emory.edu/uploads/Mentoring%20Guide_Faculty_final.pdf>
  - Student Guide: <http://www.gs.emory.edu/uploads/professional-development/Mentoring%20Guide_Student_final.pdf>

- The Council of Graduate Schools has published a “Quick Start Guide for Great Mentoring in Graduate School”, with a focus on the student perspective
  - <http://cgsnet.org/ckfinder/userfiles/files/CGS_OPS_Mentoring2016.pdf>
- The University of Minnesota has developed a series of short, useful handouts surrounding graduate mentoring, and conflict management.
  - Success for faculty mentors: <http://www.sos.umn.edu/assets/pdf/advising/SUCCESSMentors.pdf>
  - Preparing for difficult conversations: <http://www.sos.umn.edu/assets/pdf/advising/SUCCESSMentors.pdf>
  - Toxic Behavior: <http://www.sos.umn.edu/assets/pdf/advising/SUCCESSMentors.pdf>
  - Dealing with difficult behavior: <http://www.sos.umn.edu/assets/pdf/advising/SUCCESSMentors.pdf> <http://www.sos.umn.edu/assets/pdf/Students/tips.pdf>
  - Active Bystanders: <http://www.sos.umn.edu/assets/pdf/Active%20Bystanders.pdf>
- The University of Illinois has developed a toolkit for mentoring graduate students with many useful guides and tools
  - <http://www.grad.illinois.edu/faculty-staff/toolkits/mentor>
- The University of Hawai‘i at Mānoa as developed a faculty guide for mentoring graduate students.
  - <http://www2.hawaii.edu/~gurdal/grad/Mentoring_Guide.pdf>
- The Vanderbilt University Center for Teaching mentoring guide
  - <https://cft.vanderbilt.edu/guides-sub-pages/mentoring-graduate-students/#what_is>
- The Million Women Mentors, Advancing Women and Girls in Stem Careers Through Mentors, Mentoring Guide
  - <https://www.bp.com/content/dam/bp-country/en_us/PDF/Mentor-Guide-v4.pdf>
- At the University of Colorado Boulder, we have developed a short mentoring best practices document for the Ecology and Evolutionary Biology department that is included in this supplemental material.

***Other Recommended Resources for Improving Mentoring in Academia***

Books:

- *Making the Right Moves: A Practical Guide to Scientific Management for Postdocs and New Faculty*, Burroughs Wellcome Fund and Howard Hughes Medical Institute
- *At the Helm, a Laboratory Navigator,* Kathy Barker
- *Entering Mentoring: A Seminar to Train a New Generation of Scientists:* Howard Hughes Medical Institute Professors Program, Jo Handelsman, Christine Pfund, Sarah Miller Lauffer, Christine Maidl Pribbenow.
- *Faculty Success through Mentoring: a Guide for Mentors, Mentees, and Leaders,* Carole J. Bland, Anne L. Taylor, S. Lynn Shollen, Anne Marie Weber-Main, Patricia A. Mulcahy

Papers:

- *Top Ten Tips for Mentors:* Science, 2010, P. S. Clifford, J. M. Lakoski
- *Be a Good Mentor,* Inside Higher Ed, 2013, C. E. Ball
- *Characteristics of Successful and Failed Mentoring Relationships: A Qualitative Study Across Two Academic Health Centers,* Academic Medicine, 2013 (88:1:82-89), Straus et al.
- *Twelve tips for developing effective mentors,* Medical Teacher, 2006 (28:5), S. Ramani, L. Gruppen, E. K. Kachur

***Corporate Resources for Improving Mentoring***

Business Mentoring Guides:

- *Mentoring Guide,* Center for Health Leadership & Practice
  - <http://www.rackham.umich.edu/downloads/more-mentoring-guide-for-mentors.pdf>
- *Mentoring Guide,* Career Connect
  - <https://cdn2.sph.harvard.edu/wp-content/uploads/sites/31/2015/10/Mentoring_Guide.pdf>
- *Mentoring Program,* US Department of Energy, Office of Learning and Workforce Development
  - <https://energy.gov/sites/prod/files/2014/11/f19/DOE_Mentoring%20Guidance%20%20Pgrm%20Plan2_0.pdf>
- *Mentoring Program Guidelines,* Women’s Business Forum
  - <http://www.womensbusinessforum.org/assets/Mentoring-Documents/WBF-MentorGuide.pdf>
- *Introduction to Mentoring: A Guide for Mentors and Mentees,* American Psychological Association
  - [*http://www.apa.org/education/grad/mentoring.aspx*](http://www.apa.org/education/grad/mentoring.aspx)
- *Mentoring Basics- A Mentor’s Guide to Success,* NCWIT Mentoring-in-a-box: Technical Women at Work
  - [*https://www.bc.edu/content/dam/files/centers/cwf/individuals/pdf/MentorGuide.pdf*](https://www.bc.edu/content/dam/files/centers/cwf/individuals/pdf/MentorGuide.pdf)
- *Best Practices: Mentoring,* United States Office of Personnel Management
  - [*https://www.opm.gov/policy-data-oversight/training-and-development/career-development/bestpractices-mentoring.pdf*](https://www.opm.gov/policy-data-oversight/training-and-development/career-development/bestpractices-mentoring.pdf)
- *Mentoring Guide,* Xerox-ABI Online Community and Mentoring Project
  - <https://anitab.org/wp-content/uploads/2014/01/MENTORING-GUIDE.pdf>
- *Mentoring Program Guidelines,* Accredited in Business Valuation, AICPA
  - [*https://www.aicpa.org/content/dam/aicpa/interestareas/forensicandvaluation/membership/downloadabledocuments/abv-mentor-program-guidelines.pdf*](https://www.aicpa.org/content/dam/aicpa/interestareas/forensicandvaluation/membership/downloadabledocuments/abv-mentor-program-guidelines.pdf)
- *Mentoring Guide,* Grads of Life
  - [*https://gradsoflife.org/wp-content/uploads/2017/06/Mentoring-Guide.pdf*](https://gradsoflife.org/wp-content/uploads/2017/06/Mentoring-Guide.pdf)
- *Making Mentoring Work,* Nationwide, Sarah Dinolfo and Julie S. Nugent
  - [*http://www.catalyst.org/system/files/Making_Mentoring_Work.pdf*](http://www.catalyst.org/system/files/Making_Mentoring_Work.pdf)
- *Guide for Mentors,* NBAA Mentoring Network
  - [*https://www.nbaa.org/prodev/mentoring/nbaa-mentor-guide.pdf*](https://www.nbaa.org/prodev/mentoring/nbaa-mentor-guide.pdf)
- *Guide to Mentoring,* Sage Business Navigators
  - [*https://www.sage.com/en-gb/blog/wp-content/uploads/sites/10/2017/08/business-mentoring.pdf*](https://www.sage.com/en-gb/blog/wp-content/uploads/sites/10/2017/08/business-mentoring.pdf)

Books:

- *The Five Dysfunctions of a Team*, Patrick Lencioni
- *Difficult Conversations: How to Discuss What Matters Most*, Douglas Stone, Bruce Patton, Sheila Heen.
- *The Mentoring Manual: Your Step by Step Guide to Being a Better Mentor,* Julie Starr
- *Mentoring 101, What Every Leader Needs to Know,* John Maxwell
- *The Elements of Mentoring, Brad Johnson and Charles Rideley*
- *Coaching and Mentoring: How to Develop Top Talent and Achieve Stronger Performance,* Harvard Business Essentials.
- *How Did I not See this Coming: a New Manager’s Guide to Avoiding Total Disaster,* Katy Tynan
- *Modern Mentoring,* Randy Emelo
- *Business Mentoring: Communication Styles Instrument and Guide for Program Managers,* Rene Petrin
- *Creating a Successful Business Mentoring Relationship, Training, Tips, and Tricks,* Rene Petrin
- *Group Mentoring Manual for Mentors,* Rene Petrin

Papers:

- *Flourishing Enterprise,* International Society for Organizational Development and Change, by Pawan Tahilramani
- *Mentoring Perfection in Modern Enterprises Conditions: Practical Recommendations,* American Journal of Applied Sciences, 2014, Alifiya Rafisovna Masalimova et al.
- *What Google Learned from its quest to build the perfect team,* New York Times Magazine, 2016, Charles Duhigg
- *Leadership, Team Building, and Team Member Characteristics in High Performance Project Teams,* Engineering Management Journal, 2002, Anthony P. Ammeter and Janet M. Dukerich
- *Mentoring the Next Generation for Innovation in Today’s Organization,* Journal of Strategic Leadership, 2014, Teresa M. Moon
- *The Five Keys to a Successful Google Team,* re:Work, 2015, Julia Rozovsky
- *Seven Tips for Finding a Great Mentor,* Inc., Jamie Walters

***Mentoring Course***

In 2016, we developed a mentoring course in the Ecology and Evolutionary Biology department for graduate students and postdocs. In Appendix S3, we present the course syllabus and a start-up guide so this can be adopted by other programs.

***Appendix S5***

**Mentoring survey questions**

Q1 We're interested in the role mentoring plays in academic success and if/how academics are trained in mentoring. Please reflect on your experiences as both mentor and mentee. If you have not been a mentor, think about what kind of mentor you would be. After collecting a little general information about you, this online survey consists of approximately 30 short answer and multiple choice questions and should take 15-20 minutes to complete. All questions are in English. There are no known risks or discomforts associated with this survey.  Your responses will help us understand attitudes towards mentoring and common mentoring practices in STEM fields. Taking part in this survey is completely voluntary. If you choose to take the survey you can withdraw at any time.  Your responses will be kept strictly confidential and digital data will be stored in secure computer files. Other than basic demographic data (age, gender, etc.), no identifying information will be collected. Any report of this research that is made available to the public will not include any individual information by which you could be identified.

If you have questions or want a copy or summary of this study’s results, please contact Sierra Love Stowell, [sierra.lovestowell@colorado.edu](mailto:sierra.lovestowell@colorado.edu).

Clicking the “>>” button below indicates that you are 18 years of age or older, and indicates your consent to participate in this survey.

Q2 Tell us about yourself:

Q3 Which label best describes your current role?

- Undergraduate student (1)
- Graduate student - master's (7)
- Graduate student - PhD (2)
- Postdoc (3)
- Faculty (4)
- Administrator (5)
- Other (please describe) (6) ____________________

Q4 How long have you been in your current role?

- < 1 year (1)
- 1-3 years (2)
- 4-6 years (3)
- > 6 years (4)

Q5 What kind of institution do you attend or work for?

- Primary or secondary education (1)
- Community or junior college (2)
- Small liberal arts college or university (3)
- Research-oriented university (4)
- Teaching-oriented university (5)
- Government agency (6)
- Other (please describe) (7) ____________________

Q6 Where is your home institution located (country or region)?

Q7 What is your general field of study?

Q8 How old are you? Please type "Choose not to respond" if you prefer not answer this question.

Q9 What is your gender? Please type "Choose not to respond" if you prefer not answer this question.

Q10 What is your race or ethnicity? Please type "Choose not to respond" if you prefer not answer this question.

Q11 How many hours do you spend mentoring students/volunteers/employees each week? Mentoring may include one-on-one meetings, group meetings, email communication, etc.

- None (7)
- 1-3 (2)
- 4-6 (3)
- 7-9 (4)
- 10-12 (5)
- >12 (6)

Q12 How many students/volunteers/employees do you currently mentor (hours/week)?

- None (1)
- 1-2 (2)
- 3-4 (3)
- 5-6 (4)
- >6 (5)

Q13 Have you ever participated in any kind of training in mentoring?

- No (1)
- Yes (please describe) (2) ____________________

Q14 How much, if any, formal training have you received in mentoring?

- A great deal (1)
- A lot (2)
- A moderate amount (3)
- A little (4)
- None at all (5)

Q15 Have you ever written a mentoring statement or philosophy?

- No (1)
- Yes (2)

Q16 Please list three qualities you think are important in a mentor.

Q17 Please list three qualities that make someone a poor mentor.

Q18 Please list three qualities you think are important in a mentee.

Q19 Please list three qualities that make someone a poor mentee.

Q20 How important are the following qualities in a mentor?

|  | Extremely important (1) | Very important (2) | Moderately important (3) | Slightly important (4) | Not at all important (5) |
| --- | --- | --- | --- | --- | --- |
| Accessibility and responsiveness (1) |  |  |  |  |  |
| Knowledge of institutional procedures and regulations (2) |  |  |  |  |  |
| Goal-oriented (3) |  |  |  |  |  |
| Experience in your field of work/study (4) |  |  |  |  |  |
| "Connected" (5) |  |  |  |  |  |
| Empathy (6) |  |  |  |  |  |
| Consistency and fairness (7) |  |  |  |  |  |
| Patience (8) |  |  |  |  |  |
| Honesty (9) |  |  |  |  |  |
| "Savviness" (10) |  |  |  |  |  |
| Open-mindedness (11) |  |  |  |  |  |

Q21 Which statements best describes a "mentor"?

|  | Not at all (1) | Slightly (2) | Somewhat (3) | Perfectly (4) |
| --- | --- | --- | --- | --- |
| Person with career experience willing to share their knowledge (1) |  |  |  |  |
| Person who gives emotional and moral encouragement (2) |  |  |  |  |
| Person who gives specific feedback on one’s performance (3) |  |  |  |  |
| Person to whom one is apprenticed (4) |  |  |  |  |
| Person who provides information about opportunities and aid in obtaining them (12) |  |  |  |  |
| The kind of person one should be to be an academic or a professional scientist. (14) |  |  |  |  |

Q22 How important are the following qualities in a mentee?

|  | Extremely important (1) | Very important (2) | Moderately important (3) | Slightly important (4) | Not at all important (5) |
| --- | --- | --- | --- | --- | --- |
| Goal-oriented (1) |  |  |  |  |  |
| Resilience, grit, toughness (2) |  |  |  |  |  |
| Honesty (3) |  |  |  |  |  |
| Patience (4) |  |  |  |  |  |
| Independence (5) |  |  |  |  |  |
| Team player (6) |  |  |  |  |  |
| Open-mindedness (7) |  |  |  |  |  |
| Creativity (8) |  |  |  |  |  |
| Technical skills (9) |  |  |  |  |  |
| Communication skills (10) |  |  |  |  |  |
| Reliability (11) |  |  |  |  |  |

Q23 How important are the following qualities in the mentor-mentee relationship?

|  | Extremely important (1) | Very important (2) | Moderately important (3) | Slightly important (4) | Not at all important (5) |
| --- | --- | --- | --- | --- | --- |
| Honesty (1) |  |  |  |  |  |
| Directness (2) |  |  |  |  |  |
| Regular meetings (3) |  |  |  |  |  |
| Flexibillity (4) |  |  |  |  |  |
| Trust (5) |  |  |  |  |  |
| Respect (6) |  |  |  |  |  |
| Hierarchical (7) |  |  |  |  |  |
| Critical (8) |  |  |  |  |  |
| Reflective (9) |  |  |  |  |  |
| Empathetic (10) |  |  |  |  |  |
| Personal rapport or chemistry (11) |  |  |  |  |  |

Q24 How important are the following factors in graduate school retention and completion rates? (Consider the current status, rather than your ideal scenario.)

|  | Extremely important (1) | Very important (2) | Moderately important (3) | Slightly important (4) | Not at all important (5) |
| --- | --- | --- | --- | --- | --- |
| Departmental social climate (1) |  |  |  |  |  |
| Institutional social climate (2) |  |  |  |  |  |
| Financial support for stipends and living expenses (3) |  |  |  |  |  |
| Financial support for research (4) |  |  |  |  |  |
| Teaching requirements (5) |  |  |  |  |  |
| Coursework requirements (6) |  |  |  |  |  |
| Exam requirements (e.g. preliminary, comprehensive, defense exams) (7) |  |  |  |  |  |
| Mentoring from thesis advisor (8) |  |  |  |  |  |
| Mentoring from thesis committee (9) |  |  |  |  |  |
| Informal mentoring (10) |  |  |  |  |  |
| Institutional resources (e.g. equipment, space, training) (11) |  |  |  |  |  |

Q25 In your opinion or experience, how important are the following factors in faculty hiring and tenure decisions for a typical faculty position with a combination of research, teaching, and service? (Consider the current status, rather than your ideal scenario.)

|  | Extremely important (1) | Very important (2) | Moderately important (3) | Slightly important (4) | Not at all important (5) |
| --- | --- | --- | --- | --- | --- |
| Research productivity: grants (1) |  |  |  |  |  |
| Research productivity: papers (2) |  |  |  |  |  |
| Research productivity: presentations (3) |  |  |  |  |  |
| Research productivity: awards (4) |  |  |  |  |  |
| Teaching undergraduates (5) |  |  |  |  |  |
| Teaching graduate students (6) |  |  |  |  |  |
| Teaching awards (7) |  |  |  |  |  |
| Mentoring graduate students (8) |  |  |  |  |  |
| Mentoring undergraduates (9) |  |  |  |  |  |
| Mentoring postdocs (10) |  |  |  |  |  |
| Mentoring awards (11) |  |  |  |  |  |
| Departmental service (12) |  |  |  |  |  |
| Institutional service (13) |  |  |  |  |  |
| Outreach (14) |  |  |  |  |  |

Q26 As a mentee, have you experienced poor mentoring?

- No (1)
- Rarely (2)
- Frequently (3)

Q27 As a mentor, have you ever felt that you were mentoring poorly?

- I have not been a mentor (1)
- No (2)
- Rarely (3)
- Frequently (4)

Q28 Have you ever had a conflict with a mentor or mentee?

- Yes, but I would consider it a minor conflict (1)
- Yes, and I would consider it major conflict (2)
- No (3)

Q29 As a mentee, has a breakdown in the mentoring relationship ever affected you in the following areas?

|  | Never (1) | Slightly (2) | Moderately (3) | Greatly (4) | Not applicable (5) |
| --- | --- | --- | --- | --- | --- |
| Degree completion (1) |  |  |  |  |  |
| Research productivity (2) |  |  |  |  |  |
| Mental health (3) |  |  |  |  |  |
| Financial stability (4) |  |  |  |  |  |

Q30 As a mentor, has a breakdown in the mentoring relationship ever affected you in the following areas?

|  | Never (1) | Slightly (2) | Moderately (3) | Greatly (4) | Not applicable (5) |
| --- | --- | --- | --- | --- | --- |
| Degree completion, tenure, or promotion (1) |  |  |  |  |  |
| Research productivity (2) |  |  |  |  |  |
| Mental health (3) |  |  |  |  |  |
| Financial stability (4) |  |  |  |  |  |

Q31 What factors or behaviors by the mentee contribute to a breakdown in the mentoring relationship?

Q32 What factors or behaviors by the mentor contribute to a breakdown in the mentoring relationship?

Q33 How important are the following factors in developing a mentoring style?

|  | Extremely important (1) | Very important (2) | Moderately important (3) | Slightly important (4) | Not at all important (5) |
| --- | --- | --- | --- | --- | --- |
| Your experience as a mentee (1) |  |  |  |  |  |
| Training in mentoring (2) |  |  |  |  |  |
| Advice from peers (3) |  |  |  |  |  |
| Advice from your former mentees (4) |  |  |  |  |  |
| Pamphlets, articles, and books (5) |  |  |  |  |  |
| Learning by doing (6) |  |  |  |  |  |

Q34 If you were to participate in mentoring training, what activities or resources would you find useful (select all that apply)?

- Personality and strengths inventories (1)
- Conflict resolution (2)
- Communication best practices (3)
- Hiring practices (4)
- Holding effective meetings (5)
- Consultation (6)
- Other (please describe) (7) ____________________

Q35 Is there anything else you would like to express about the role of mentoring and/or training in mentoring?

Q36 Thank you very much for your time.

***Appendix S6***

**Qualtrics Mentoring Survey Summary Results**

Number of respondents who completed >53% of survey:

235

Gender

| **Response** | **Female** | **Male** | **Non-binary / No response** |
| --- | --- | --- | --- |
| **Count** | 130 | 100 | 5 |

Age

| **Response** | **18-22** | **23-27** | **28-32** | **33-37** | **38-42** | **43-47** | **48-52** | **53-57** | **58-62** | **no response** |
| --- | --- | --- | --- | --- | --- | --- | --- | --- | --- | --- |
| Count | 21 | 62 | 64 | 36 | 19 | 7 | 8 | 8 | 6 | 4 |

Role

| **Response** | **Administrator** | **Faculty** | **Graduate student - master's** | **Graduate student - PhD** | **Postdoc** | **Undergraduate student** | **Other** |
| --- | --- | --- | --- | --- | --- | --- | --- |
| **Count** | 5 | 43 | 23 | 74 | 43 | 20 | 27 |

“Other” included staff research scientists, technicians, and assistants for universities, government agencies, and non-profit organizations

Time in position

| **Duration (years)** | **<1** | **1-3** | **4-6** | **> 6** |
| --- | --- | --- | --- | --- |
| **Count** | 44 | 100 | 55 | 36 |

Field

| **Response** | **BIO** | **CHEM** | **ECO&EVO** | **ENG** | **ENVIRO** | **MATH** | **MED** | **PHYS** | **TECH** |
| --- | --- | --- | --- | --- | --- | --- | --- | --- | --- |
| **Count** | 80 | 8 | 51 | 17 | 16 | 3 | 3 | 16 | 21 |

Race/Ethnicity/Geographic Descent

| **Response** | **Asian** | **Black or Biracial** | **Hispanic** | **Middle Eastern** | **White** | **No response** |
| --- | --- | --- | --- | --- | --- | --- |
| **Count** | 14 | 3 | 13 | 2 | 192 | 11 |

Weekly time spent mentoring

| **Time (hours/week)** | **0** | **1-3** | **4-6** | **7-9** | **10-12** | **>12** |
| --- | --- | --- | --- | --- | --- | --- |
| **Count** | 57 | 87 | 49 | 14 | 16 | 12 |

Current number of mentees

| **Response** | **0** | **1-2** | **3-4** | **5-6** | **>6** |
| --- | --- | --- | --- | --- | --- |
| **Count** | 63 | 84 | 41 | 21 | 26 |

Received training in mentoring? No: 162 Yes: 73

How much training in mentoring?

| **Response** | **None** | **A little** | **Moderate** | **A lot** |
| --- | --- | --- | --- | --- |
| **Count** | 137 | 65 | 23 | 9 |

Types of training

Mentoring/being mentored 13

Formal coursework 9

Long workshop (HHMI SI, Entering Mentoring, etc) 12

Short workshop 9

Multiple workshops 11

TA or tutor training 7

Web training 3

Military or sports 3

Ever written a mentoring statement? No: 169 Yes: 66

Three words to describe a good mentor (709 words) (textalyser.net)

| **Word** | **Occurrences** | **Frequency** |
| --- | --- | --- |
| patience | 85 | 12% |
| listening | 41 | 5.80% |
| empathy | 34 | 4.80% |
| communication | 27 | 3.80% |
| knowledgeable | 25 | 3.50% |
| caring | 20 | 2.80% |
| honest | 20 | 2.80% |
| openness | 19 | 2.70% |
| encouraging | 17 | 2.40% |
| experienced | 16 | 2.30% |

Three words to describe a bad mentor (721 words)

| **Word** | **Occurrences** | **Frequency** |
| --- | --- | --- |
| unavailable | 44 | 6.10% |
| impatient | 44 | 6.10% |
| self-absorbed | 38 | 5.30% |
| uncommunicative | 30 | 4.20% |
| disinterested | 23 | 3.20% |
| arrogant | 19 | 2.60% |
| disorganized | 16 | 2.20% |
| inflexible | 13 | 1.80% |
| egotistical | 12 | 1.70% |

*similar words come up in responses to Question 32 (what factors or behaviors by the mentor contribute to a breakdown in the mentoring relationship?)

Three words to describe a good mentee (695)

| **Word** | **Occurrences** | **Frequency** | **Rank** |
| --- | --- | --- | --- |
| Hard working, work ethic | 42 | 6% | 1 |
| Good listener, willing to listen | 40 | 5.80% | 2 |
| Open-minded | 30 | 4.30% | 3 |
| Communicator | 29 | 4.20% | 4 |
| motivated | 26 | 3.70% | 5 |
| learner | 25 | 3.60% | 6 |
| Open to new ideas, experiences, advice | 22 | 3.20% | 7 |
| Interested in topic, in learning | 21 | 3% | 8 |
| Curious | 20 | 2.90% | 9 |
| Self-aware, self-motivated, self-reflective | 20 | 2.90% | 9 |

Three words to describe a bad mentee (679)

| **Word** | **Occurrences** | **Frequency** |  | **Rank** |
| --- | --- | --- | --- | --- |
| lazy, unwilling to work, poor work-ethic | 55 | 8.10% |  | 1 |
| disinterest, uninterested | 28 | 4.10% |  | 2 |
| apathetic, unempathetic | 26 | 3.80% |  | 3 |
| uncommunicative, poor communication | 26 | 3.80% |  | 3 |
| narrow-minded, close-minded | 24 | 3.50% |  | 4 |
| arrogant | 23 | 3.40% |  | 5 |
| closed, rigid | 22 | 3.20% |  | 6 |
| entitled | 18 | 2.70% |  | 7 |
| impatient | 18 | 2.70% |  | 7 |
| fails to accept critique or advice | 17 | 2.50% |  | 8 |

*similar words come up in responses to Question 31 (what factors or behaviors by the mentee contribute to a breakdown in the mentoring relationship?)

Q20: How important are the following qualities in a mentor?

|  | **Extremely important** | **Very important** | **Moderately important** | **Slightly important** | **Not at all important** |
| --- | --- | --- | --- | --- | --- |
| Accessibility and responsiveness | **56.1** | 35.4 | 7.6 | 0.4 | 0.4 |
| Knowledge of institutional procedures and regulations | 14.3 | **40.4** | 35.4 | 9.0 | 0.9 |
| Goal-oriented | 20.6 | **48.0** | 24.7 | 6.3 | 0.4 |
| Experience in your field of work/study | 37.7 | **42.6** | 17.0 | 1.8 | 0.9 |
| "Connected" | 15.2 | 34.1 | **40.8** | 8.5 | 1.3 |
| Empathy | **49.8** | 33.2 | 13.0 | 3.6 | 0.4 |
| Consistency and fairness | **47.1** | 41.3 | 10.3 | 0.9 | 0.4 |
| Patience | **62.3** | 29.1 | 6.3 | 1.3 | 0.9 |
| Honesty | **62.8** | 31.8 | 4.5 | 0.4 | 0.4 |
| "Savviness" | 9.9 | 34.1 | **40.8** | 12.1 | 2.7 |
| Open-mindedness | **49.3** | 5.4 | 10.8 | 39.5 | 0.4 |


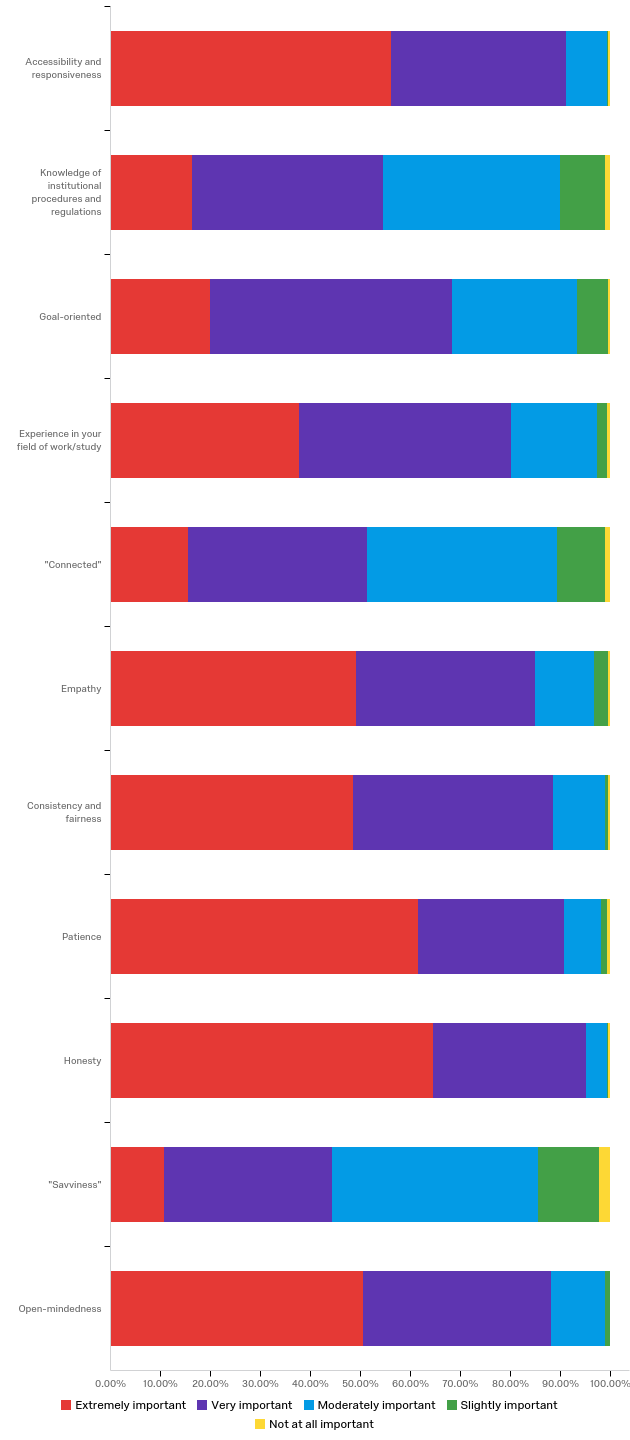


Q21: Which statements best describe a mentor?

|  | Perfectly | Somewhat | Slightly | Not at all |
| --- | --- | --- | --- | --- |
| Person with career experience willing to share | **50.7** | 40.8 | 8.1 | 0.4 |
| Person who gives emotional and moral encouragement | 30.5 | **52.5** | 15.2 | 1.8 |
| Person who gives specific feedback on one's performance | 33.3 | **49.5** | 15.3 | 1.8 |
| Person to whom one is apprenticed | 18.5 | **40.5** | 30.2 | 10.8 |
| Person who provides information about opportunities and aid in obtaining them | 23.4 | **50.0** | 26.1 | 0.5 |
| The kind of person one should be to be an academic or professional scientist | 24.4 | 36.7 | 26.7 | 12.2 |


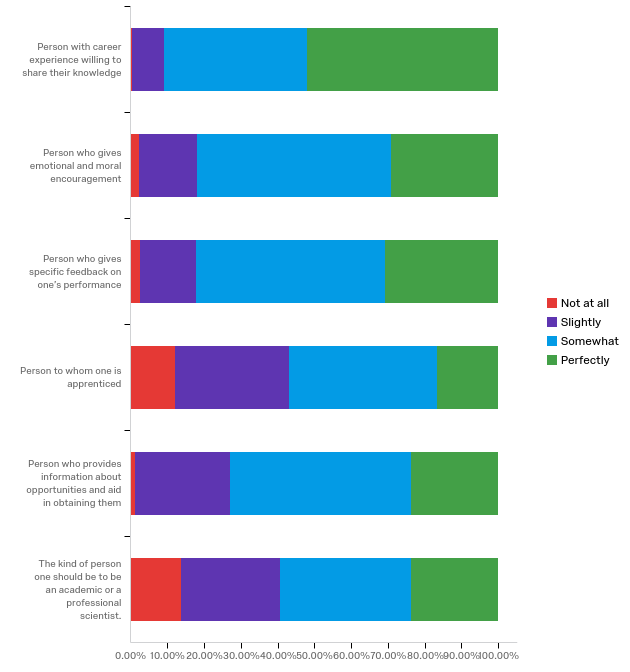


Q22: How important are the following qualities in a mentee?

|  | Extremely important | Very important | Moderately important | Slightly important | Not at all important |
| --- | --- | --- | --- | --- | --- |
| Goal-oriented | 30.5 | **43.2** | 22.3 | 3.6 | 0.5 |
| Resilience, grit, toughness | **40.9** | 35.0 | 19.1 | 3.6 | 1.4 |
| Honesty | **58.7** | 6.8 | 4.7 | 29.4 | 0.4 |
| Patience | **47.5** | 38.4 | 11.9 | 1.8 | 0.5 |
| Independence | 26.9 | **50.2** | 17.8 | 3.7 | 1.4 |
| Team player | 30.9 | **42.7** | 20.9 | 4.1 | 1.4 |
| Open-mindedness | **51.8** | 42.7 | 4.5 | 0.5 | 0.5 |
| Creativity | 25.1 | **42.0** | 27.9 | 3.2 | 1.8 |
| Technical skills | 5.9 | 31.5 | **39.7** | 18.3 | 4.6 |
| Communication skills | 36.1 | **45.2** | 16.9 | 1.4 | 0.5 |
| Reliability | **63.5** | 32.9 | 2.3 | 0.9 | 0.5 |


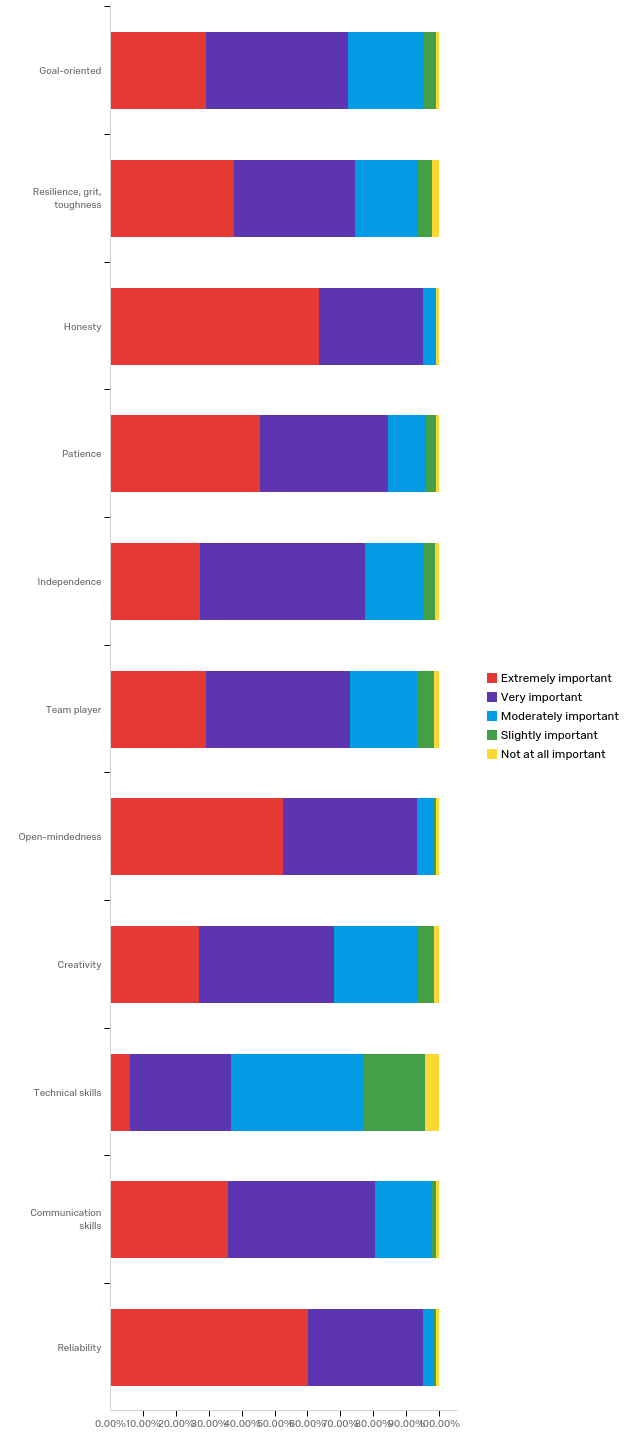


Q23: How important are the following qualities in the mentor-mentee relationship?

|  | Extremely important | Very important | Moderately important | Slightly important | Not at all important |
| --- | --- | --- | --- | --- | --- |
| Honesty | **71.1** | 6.0 | 1.7 | 20.9 | 0.4 |
| Directness | **47.1** | 39.8 | 11.8 | 0.9 | 0.5 |
| Regular meetings | 21.7 | **39.8** | 29.0 | 6.8 | 2.7 |
| Flexibility | 28.1 | **49.3** | 20.8 | 1.4 | 0.5 |
| Trust | **72.3** | 6.0 | 1.3 | 20.0 | 0.4 |
| Respect | **86.4** | 10.9 | 1.8 | 0.5 | 0.5 |
| Hierarchical | 2.3 | 9.5 | **31.4** | 29.5 | 27.3 |
| Critical | 6.3 | 32.6 | **34.4** | 20.8 | 5.9 |
| Reflective | 26.4 | **45.0** | 25.0 | 2.7 | 0.9 |
| Empathetic | 32.6 | **39.8** | 23.5 | 3.2 | 0.9 |
| Personal rapport or chemistry | 19.5 | **39.1** | 31.4 | 9.1 | 0.9 |


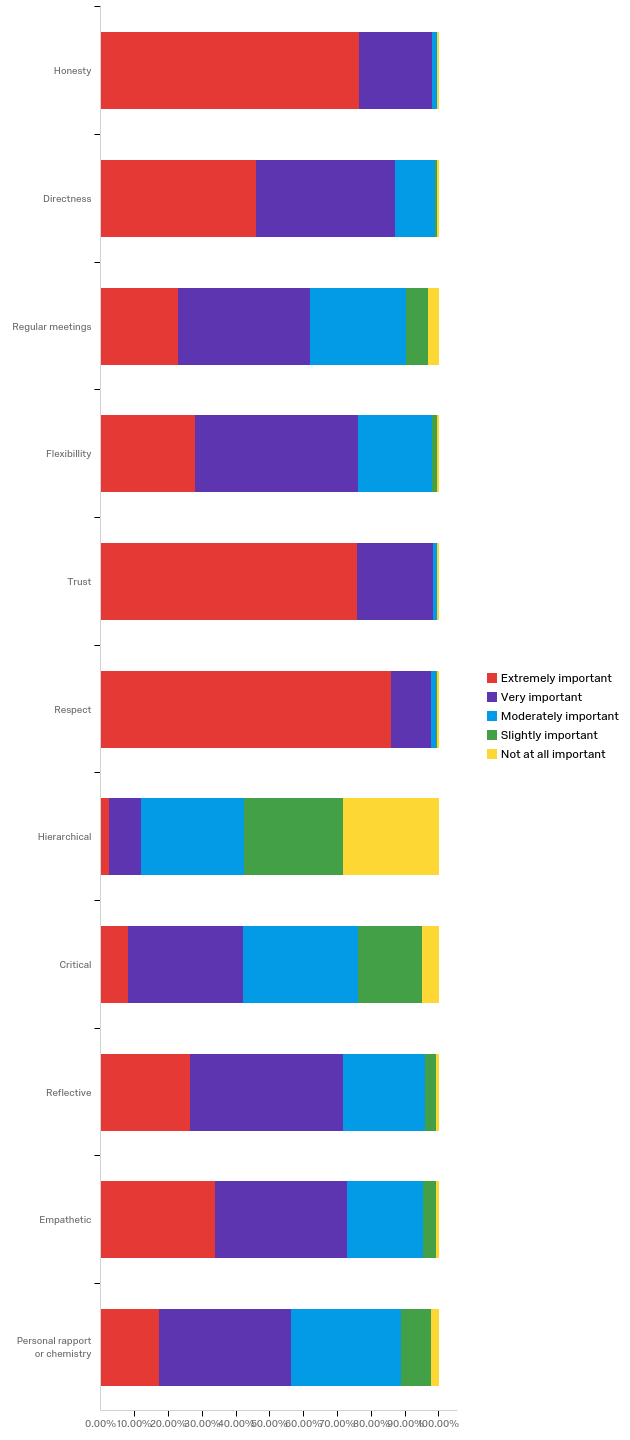


Q24: How important are the following factors in graduate school retention and completion rates?

|  | Extremely important | Very important | Moderately important | Slightly important | Not at all important |
| --- | --- | --- | --- | --- | --- |
| Departmental social climate | 27.4 | **49.5** | 17.9 | 4.2 | 0.9 |
| Institutional social climate | 10.8 | 27.4 | **40.6** | 17.9 | 3.3 |
| Financial support for stipends and living expenses | **52.8** | 9.8 | 6.8 | 30.2 | 0.4 |
| Financial support for research | **48.1** | 39.2 | 10.4 | 1.9 | 0.5 |
| Teaching requirements | 8.5 | 27.0 | **40.8** | 19.9 | 3.8 |
| Coursework requirements | 7.1 | 23.6 | **46.2** | 18.4 | 4.7 |
| Exam requirements | 6.6 | 30.2 | **42.0** | 19.3 | 1.9 |
| Mentoring from advisor | **68.9** | 25.9 | 4.2 | 0.5 | 0.5 |
| Mentoring from committee | 14.2 | **40.1** | 29.7 | 13.7 | 2.4 |
| Informal mentoring | 22.6 | **46.2** | 25.5 | 3.8 | 1.9 |
| Institutional resources | 23.4 | 9.8 | 24.7 | **38.7** | 3.4 |


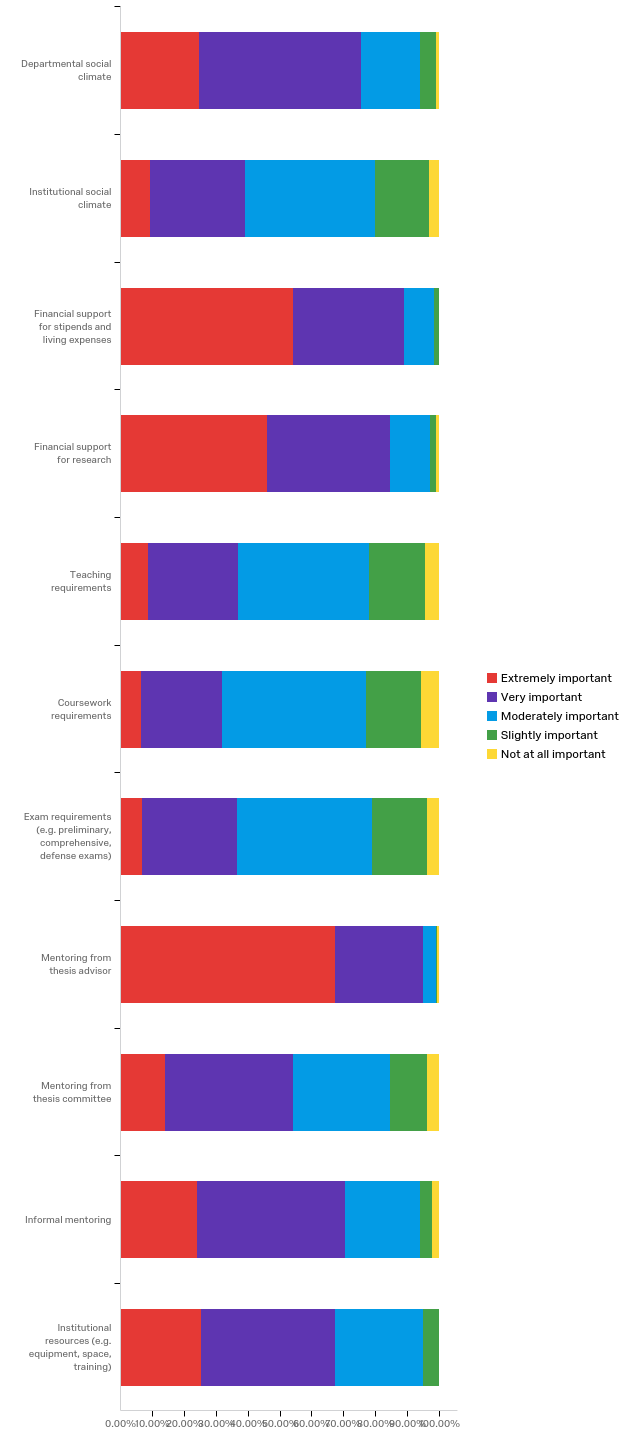


Q25: How important are the following factors in faculty hiring and tenure decisions for a typical faculty position with a combination of research, teaching, and service?

|  | **Extremely important** | **Very important** | **Moderately important** | **Slightly important** | **Not at all important** |
| --- | --- | --- | --- | --- | --- |
| Research productivity: grants | **56.3** | 31.6 | 10.2 | 1.5 | 0.5 |
| Research productivity: publications | **65.0** | 27.2 | 6.3 | 0.5 | 1.0 |
| Research productivity: presentations | 12.1 | 34.5 | **41.3** | 10.7 | 1.5 |
| Research productivity: awards | 11.7 | 30.6 | **34.0** | 20.9 | 2.9 |
| Teaching undergraduates | 17.5 | 21.4 | **29.6** | 22.3 | 9.2 |
| Teaching graduate students | 10.7 | 27.7 | **33.5** | 19.4 | 8.7 |
| Teaching awards | 1.9 | 8.3 | 29.1 | **41.7** | 18.9 |
| Mentoring graduate students | 18.9 | **31.1** | 29.1 | 12.6 | 8.3 |
| Mentoring undergraduates | 13.1 | 17.0 | **28.6** | 24.3 | 17.0 |
| Mentoring postdocs | 11.7 | 24.3 | **26.2** | 24.8 | 13.1 |
| Mentoring awards | 2.4 | 6.8 | 27.7 | **32.5** | 30.6 |
| Departmental service | 5.3 | 23.3 | **42.2** | 24.8 | 4.4 |
| Institutional service | 4.9 | 15.5 | **39.8** | 31.6 | 8.3 |
| Outreach | 6.8 | 18.0 | 29.6 | **31.6** | 14.1 |


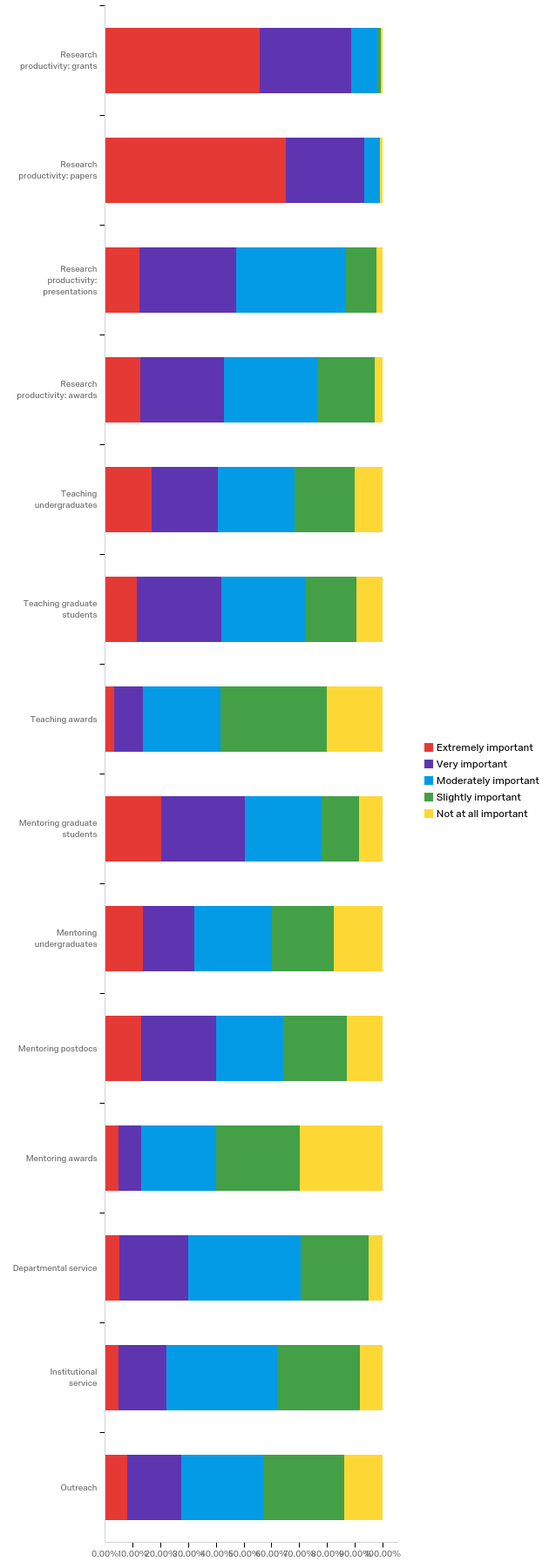


Q26: As a mentee, have you experienced poor mentoring?

Frequently: 38.8% (83)

Rarely: 49.1% (105)

Never: 12.1% (26)

Q27: As a mentor, have you ever felt you mentoring poorly?

Never mentored: 13.1% (28)

Frequently: 16.8% (36)

Rarely: 61.2% (131)

Never: 8.9% (19)

Q28: Have you ever had a conflict with a mentor or mentee?

No: 26.0% (56)

Yes, but I would consider it a minor conflict: 47.9% (103)

Yes, and I would consider it a major conflict: 26.0% (56)

Q29: As a mentee, has a breakdown in the mentoring relationship ever affected you in the following areas?

|  | Greatly | Moderately | Slightly | Never |
| --- | --- | --- | --- | --- |
| Degree completion | 14.4 | 9.3 | 14.4 | 61.9 |
| Research productivity | 22.1 | 21.1 | 27.1 | 29.6 |
| Mental health | 26.0 | 20.0 | 23.5 | 30.5 |
| Financial stability | 7.7 | 11.3 | 14.9 | 66.0 |


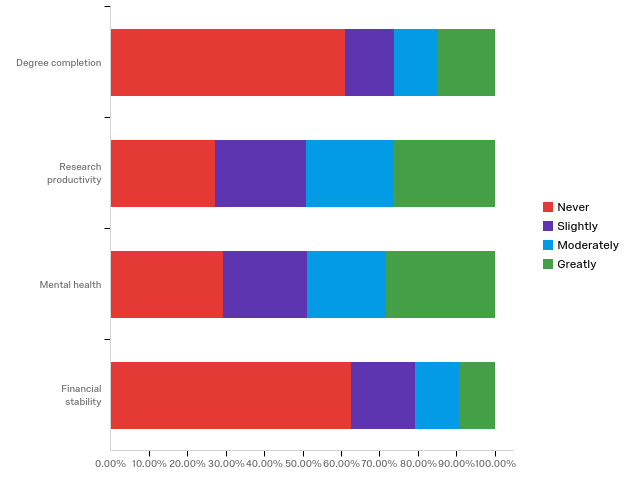


Q30: As a mentor, has a breakdown in the mentoring relationship ever affected you in the following areas?

|  | Greatly | Moderately | Slightly | Never |
| --- | --- | --- | --- | --- |
| Degree completion | 2.0 | 4.0 | 10.0 | 84.0 |
| Research productivity | 5.1 | 19.9 | 33.3 | 41.7 |
| Mental health | 4.4 | 8.1 | 26.9 | 60.6 |
| Financial stability | 1.3 | 3.9 | 4.6 | 90.1 |


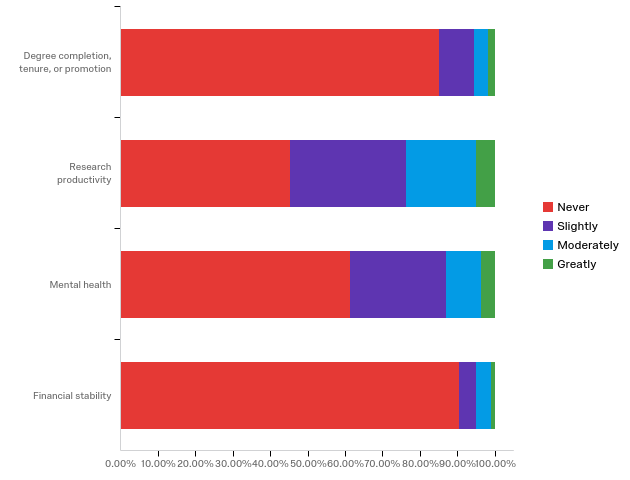


Q33: How important are the following factors in developing a mentoring style?

|  | Extremely important | Very important | Moderately important | Slightly important | Not at all important |
| --- | --- | --- | --- | --- | --- |
| Your experiences as a mentee | **53.8** | 34.8 | 8.6 | 1.9 | 1.0 |
| Training in mentoring | 8.2 | 23.2 | **35.3** | 22.7 | 10.6 |
| Advice from peers | 9.1 | 38.5 | **42.8** | 8.7 | 1.0 |
| Advice from your former mentees | 22.3 | **47.1** | 21.8 | 7.3 | 1.5 |
| Pamphlets, articles, and books | 1.4 | 11.1 | 30.4 | **41.5** | 15.5 |
| Learning by doing | **57.0** | 29.0 | 13.0 | 0.5 | 0.5 |


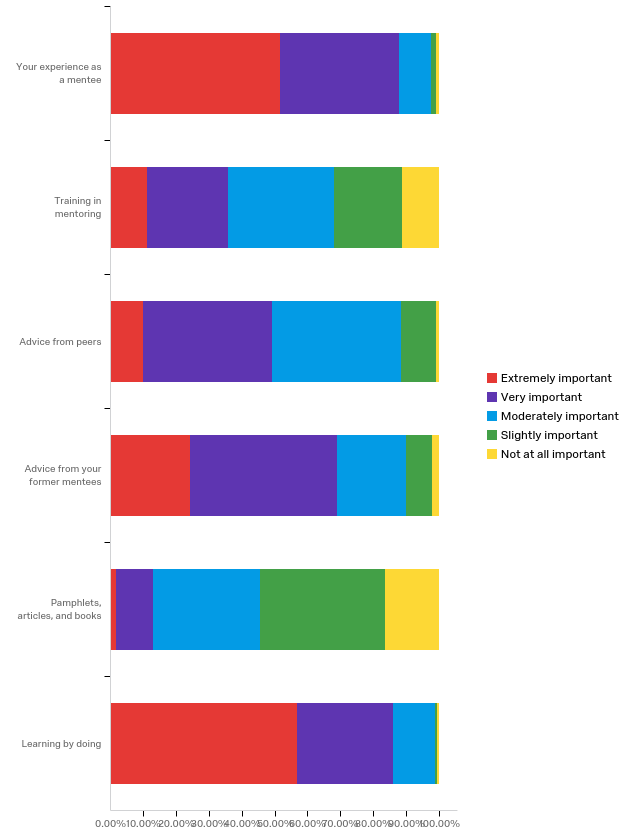


Q34: If you were to participate in mentoring training, what activities or resources would you find useful (select all that apply)?

Communication best practices: 175

Conflict resolution: 137

Holding effective meetings: 131

Personality and strengths inventories: 89

Hiring practices: 58

Consultation: 44

Other: 26

Other text:

| Addressing mental illness that affects progress (e.g., depression, anxiety) |
| --- |
| basic mentoring 101...why we mentor, what do people need when being mentored, the benefits of mentoring, etc |
| Case studies in mentoring, good and bad |
| dealing with mental health issues (while at the same time not getting embroiled with them) |
| Dealing with students who have mental challenges. |
| developing multicultural awareness |
| diversity training, how to work with students with disabilities or other health limitations |
| Empathy |
| empowerment |
| field-specific training |
| How to be approachable |
| how to offer good advice |
| Inclusivity |
| Information about types of mentors, recognition that different situations will call for different mentoring roles. |
| Institutional support and guidelines, transparency |
| knowledge of university rules and regs |
| logistical and time management while overlooking multiple projects |
| Motivating by example |
| not another train program please |
| Processes for soliciting feedback from mentees |
| Time management skills |
| Training in how people learn |
| Training in mentoring diverse students, mental health awareness, teaching rather than training: how to empower students to learn themselves, case studies of ineffective mentoring/common concerns/criticisms from mentees |
| transfiguration practice. I need to know how to disguise myself |

Q35: Is there anything else you would like to express about the role of mentoring and/or training in mentoring?

- Don't call the students or trainees "mentees." The term is not favored among the mentoring teaching community anymore.
- I have experienced severe breakdown of the mentor-mentee relationship when my advisor didn't communicate his expectations with me, then I didn't perform according to his expectations, so he stopped talking to me for 4 months. Communication is crucial, as is a stable personality.
- Mentoring is not officially taught in STEM, therefore mentors vary greatly in quality.
- Different mentees need different types of mentoring, and that can change over time (e.g. networker, coach, confidante, adviser)
- a good relationship is important
- Academia is overly reliant on what mentors think about their mentees. This affects postdocs the worst because usually there is no other supervisor than the advisor who can give a different perspective to future employers. This needs to change. There also needs to be a better grievance filing procedure. Mentors need to be told during the training that they don't own the mentees and that biased and bad references in future will not be tolerated as the institutions value their mentees as much as their mentors.
- Also important is training mentees in how to seek out and approach potential mentors
- Best mentors I have had have been honest and direct, but understanding of what mentees are experiencing (i.e. 'Do what you need to do for your health, we will make a plan to work around it to keep the research moving forward.')
- Worst mentors I have seen are honest and direct about whatever comes into their head with no consideration of their mentees experience (I.e. 'You should cut your friends out of your life if they aren't going to be professors because they have nothing to offer you').
- Each student/mentee is different and you have to be able to adjust your mentoring style accordingly. You also have be honest and realize when a student isn't suited for their position and if they can't make the decision to leave on their own, you need to end it.
- I'd also wonder for women, how many female mentors they have and if that makes a difference; especially in STEM where we see a huge drop off in women at the postdoc and faculty levels compared to undergrad and grad student level. This hasn't changed."
- Everyone should mentor, and mentor often.
- Experiencing excellent mentoring while an undergraduate is the primary reason I'm pursuing a PhD and feel supported in doing this - continuity of mentoring (still by my undergraduate advisor, now supplemented by my PhD advisor).
- For training in mentoring, a recurrent every two or three month training session would be more useful than a one time thing
- Grad students need training in mentoring so that if we become professors/bosses we don't suck at it.
- Hearing horror stories about advisor/advisee relationships from graduate students cause me to prioritize finding an advisor that I thought I could work well with over most other considerations in selecting a graduate school. Good mentors who took an interest in and encouraged my work before grad school were a lot of the reason I stayed in science.
- I attribute much of my professional success to two mentors as a graduate student, one of whom was pre-tenure faculty and a co-adviser for my PhD and the other of whom retired in my last year of school and I worked for as a TA for three years (in a position outside my department). These people had a long-term view of my career even when it was too early for me to see the bigger picture and generally were respectful and compassionate individuals. As I have moved into my faculty role since graduating last May, both have remained incredibly involved in my professional life. Since I graduated their roles have expanded more into moral support/affirmation as well, which was not so much what I was looking for while in school (then I was looking mostly for support in the form of information), and they have advised me on some stressful episodes in my new position.
- I believe to be a good mentee you need to be willing to put some blind faith in the mentor. Sometimes my mentors would tell me things (e.g., you should try this set of experiments, you should try cold-calling when you teach your class) and in the beginning I would resist and sometimes fail to act on their suggestions, which in retrospect I imagine was a frustrating experience for them. At some point I had an epiphany (I actually could describe the exact moment when this happened, and it involved me being told off by my TA supervisor!) and realized, heck, these guys know so much more than me, maybe sometimes I should listen to what they say and do it without always questioning why! And this was really when I began to love grad school, because their suggestions rarely led me to the answer, but they did provide launch points that enabled me to discover new questions and answers for myself. If anything now I am bothered by mentoring relationships where I don't perceive enough negative feedback. A mentoring relationship is about encouraging growth.
- I believe many students fail to understand that mentoring is a two-way street, not only in the need for the student to actively participate in the mentoring relationship (as opposed to passively receiving information), but also some students fail to recognize that they can be selective about their mentors. True, you choose your PhD adviser because you have common research interests. But my TA supervisor was totally outside my field and he was literally the only person who read my job applications (not saying this was a good idea) because I became so comfortable with how we delivered and received feedback, and this was a mentor I went out and found and cultivated myself after I enjoyed his class outside my department. I also came into grad school to work with a different major professor and I didn't like how the advising relationship was going so I left the department with a Master's and switched into a different department at the same institution to work with my PhD adviser who I met in the course of the bad Master's program. I also had a PhD co-adviser who I felt was too much of a micromanager and stole/took credit for many of his employees ideas and I have not worked with them since I graduated. As a student you can initiate mentoring relationships and you can terminate them.
- I am interested to see how things look on the faculty side. It is my first semester in my new role and so far my mentoring is confined to helping my TA with teaching development and job applications. I am taking on undergrad research students next semester and grad students in the fall. Like everything, I anticipate the best learning will be from watching other people's experiences and learning from my own mentors from my grad program and at my current institution.
- I am interested that more on the gender of the mentor/mentee didn't figure into the survey. I am a woman, and both my mentors in grad school were men, and I have a female mentor in my new faculty position with whom I feel like I can discuss issues like workplace bias that were harder to discuss with my truly exceptional male mentors simply because as two white guys they were less exposed to these things in their personal experience.
- I believe that mentoring, especially informal mentoring, is absolutely essential for an undergraduate college student to be prepared for post-graduation life or further education.
- I had a great mentor for my undergrad thesis and the experience helped me make the decision to pursue a career in academia. Mentors really do make, or break, those experiences. And the simple ability to give your full attention to the mentee, and do your best to help as they are learning new things, is easy but makes all the difference.
- I had poor experiences with formal mentors from 4th grade until the middle of my bachelors degree. While I could overcome social incompatibility (we'd never be friends), I cannot forgive incorrect advice. If a mentor gives a confident answer to a simple question (e.g., "will this class be offered next semester?"), they'd better be right. An uncertain answer ("I think..., but here's how you can check") is fine. But, if I later find out their sure answer was wrong, I will have difficulty trusting any later answers or advice they give, and will effectively check out of the relationship. I'd like to say a sincere apology would prevent this, but I have never received one in this situation.
- I have seen many attempts at formalizing a mentoring program and all of them failed. I think mentoring works best when there is chemistry between the two parties.
- I think it is critical that mentors not hold students/mentees back by not being open-minded to and supportive of the mentee's own research/career interests.
- Also, I think it is critical that departments reconsider to some extent the standard faculty hiring process. As a graduate student, I was privileged to sit in on some department searches, and all too often I saw that the department considered only the stereotypical factors in a faculty search (such as awards, grant productivity, publishing productivity, etc.) with little to no regard for mentoring capabilities, and certainly no regard to graduate student perspectives in mentee experiences. Departments need to de-emphasized the "earning" capabilities of a faculty hire and focus more on "giving" capabilities, in the sense of what positive values that potential faculty member can contribute to the department, particularly on behalf of student mentoring. Too often as a graduate student (ie primarily a mentee vs mentor) I experienced harshness, inattentiveness, inflexibility, and a general lack of caring for student well-being from professors toward their students; this was an epidemic throughout the department, not just a few exceptions. Departments need to realize that the true marks of what makes a department great are not the awards for research or the grant money brought in, but the formation of quality future educators/career people through quality guidance and mentoring of students. "
- I think mentoring well is challenging, and faculty rarely receive training in how to become better mentors. However, I think all graduate students and postdocs should receive mentor training, so all faculty have some mentor training prior to taking on large numbers of students.
- I think that it's an often overlooked yet essential element in undergraduate and graduate education. Both mentors and mentees lack resources that help them perform their best, and it would be cool if we could change that.
- I think that training in mentoring and managing personnel should be mandatory for all faculty.
- I understand it's (at least partially) the point of the survey, but it's very clear that faculty rarely have or are provided guidance on mentoring and therefore, it's another skill that is needed when moving into that type of position. Therefore, this survey is important. Thanks for doing whatever it is you're doing.
- I was verbally abused by two mentors early in my career and it had a significant negative impact on my professional development. I want to positively influence others' professional development. I wish I had access to mentoring training.
- I wish I had more mentoring training. I received outstanding mentoring as an undergraduate, and thought I could use my experience as a mentee to mentor others. But I found that communication was an issue, and I had a hard time maintaining a productive relationships with my undergraduates, particularly when lab work quality was poor.
- I would like to get specific training in being a good mentor to women and minority students.
- I'm skeptical of processes where an institution sees "there is a problem with how we're doing X" and concludes "we should have a formal seminar to learn how to do X". I think these seminars tend to be more or less a waste of time. I'm not sure what a better solution is, though :|
- In 4.5 years of grad school, I have yet to be part of a conversation on developing mentoring skills, despite it being the reason most of us got into research science in the first place. Perhaps its time for me to start that conversation!
- In academia it's the most important institution that exists. Mentorship and being a good mentor are to me the most important goals of my career.
- In my opinion the quality of academic work would increase if the mentoring on universities improved. I believe that the mentoring and, in general, academic environment is such that does not distinguish people with the characteristics the universities claim to seek in the entrance applications. Rather it promotes people with either prior knowledge of the environment that are prepared to tackle it before entering, or people with concerning social lives and questionable social skills.
- It is a huge part of our daily lives, yet it is never addressed specifically in our training and ignored completely in the faculty hiring process.
- It is particularly tough when the mentor or mentee is from a significantly different background than yours. Cultural awareness and understanding is not stressed enough.
- it would be nice that R1 universities considered mentoring as part of tenure or as part as a grad student's advancement in their careers.
- Mentoring can exist outside of academics
- Mentoring in academia is often not great. Just because someone is a good teacher does not make them a good mentor. An outside mediator, concerned only with graduate student & postdoc health & development may be incredibly useful. Likewise, always being mentored by two members of faculty may help.
- Mentoring is an enormously undervalued skill in tenure track faculty hires at research universities. I believe without either adequate training or at the very least putting as much emphasis on mentoring experience as publication output during hiring processes, one does a disservice to not only those more qualified for these leadership positions but also to students and future scientists.
- Mentoring is very important, especially for graduate students. During my PhD, I had a very difficult relationship with my thesis advisor because I did not feel respected or valued. He viewed graduate students as "cheap labor" and continually used us for his own projects instead of helping us develop our own. By contrast, an unofficial mentor that I became associated with treated me very well - he supported my projects and help me develop a sense of independence. He trusted me. The major difference between the two was in their personal characteristics (personality, degree of arrogance, confidence v. insecurity in their own careers), and these are things that can't be fixed by mentorship training. You can't train a senior scientist to care about their graduate students. My advice to prospective mentees would be to talk to their mentor's current students and ask what the mentor is like. I would also advise students to seek multiple official and unofficial mentors, to find someone they can trust, and stick with them.
- Mentoring should not be confined to the professor-student relationship, more peer-mentoring is required to diversify the sciences and bridge the gap for English as a second language scientists and scientists working in/coming from developing countries.
- Mentoring, like teaching, has a large component that can't be easily taught. I've seen folks with all kinds of training in teaching and mentoring, and they are horrible at both given their personalities. That said, just about anyone can improve their mentoring through classes, self-learning, and experience. I find personality tests, though seemingly standard fare in mentoring and leadership training, to be rather useless to actually use 'on the fly'...while mentoring someone.
- My advisor ignored me for the course of my entire graduate career (NEVER met with me or gave me any advice), and then abandoned me for a high-paying job in the middle of nowhere. I got a fucking divorce over this, and my mental well-being has suffered considerably.
- My advisor is somebody who always assigns tasks to both of us - not just me. He makes me feel like I am a part of his team and not like I work under him. Every meeting goes like - you try to do this and I will try to do this and we will come together next week and try to merge our results. I feel that keeps me going strong and makes me feel good about the work we are doing. :)
- My most successful mentoring relationships have always been informal. I have been a "mentee" in several "mentor" programs where a mentor was assigned, and these have usually not taught me anything. I don't think you can be "assigned" a mentor. You have to find one on your own, or they find you.
- My PhD advisor was an instructive example. He was gruff and impersonal. He explicitly had high expectations for his students I terms of time investment and productivity. He literally didn't speak to me for my first year in the lab. During this time I was great full for excellent informal mentoring from older grad students and post docs. In time, I started generating data, and my PhD advisor took notice. Ultimately, he became a very helpful mentor in writing, publishing and experimental design. But in terms of managing people he will always be a negative example! The silver lining is that I now feel like I can work with just about any anyone!
- not everyone is equipped to serve as a mentor but most can benefit from being a mentee
- One huge problem I found as a graduate student is that there are no easy ways to provide feedback to advisors about their mentoring. Feedback on my advisor's mentorship was NEVER solicited by my advisor or anyone in the department or university. This meant that providing constructive criticism and feedback to improve her mentoring abilities was awkward and could potentially have jeopardized our relationship (and therefore future letters of recommendation). Graduate students are in a vulnerable position and dependent in many ways on their advisors, and they also have no stake in the future environment of their lab. This combination means that many grad students would prefer to "suffer through" a bad advisor than "speak up" about poor mentoring/leadership... resulting in highly socially dysfunctional labs and contributing to poor mental health of graduate students.
- People with very different cultures/backgrounds from our own often think and communicate very differently than we do. The need for a training program addressing multicultural awareness has been brought up many times, and I hope that aspect can be incorporated into training for future mentors.
- There is an enormous gap in mentoring training at my R1 university. My advisor has never had any coaching in how to mentor, and she is pretty terrible at it. She refuses to listen to hints or suggestions for improvement. There is only room for criticism in her mentoring style as she 'doesn't have time for giving positive feedback'. A little encouragement goes a long way!
- This is so important! I didn't realize how poor my relationship is with my own advisor/mentor until I started taking a workshop on training grad students to mentor undergrads. Learning about how to align expectations, provide feedback, and work towards outcomes with undergrad mentees made me realize how skewed my relationship with my own mentor is, and I want to make sure I do better for mentees in the future!
- University doesn't care about bad relationships between mentor/mentee. No help can be found to resolve potential conflicts.
- You didn't ask how important a mentor has been in my professional success. Extremely!
